# Deciphering the Glycosylation Steps in the Biosynthesis of P-1894B and Grincamycin Isolated from Marine-Derived *Streptomyces lusitanus* SCSIO LR32

**DOI:** 10.3390/md22010032

**Published:** 2024-01-02

**Authors:** Hongbo Huang, Yun Zhang, Yongxiang Song, Chunyao Ling, Siyan Peng, Bo Ding, Yiwen Tao, Jianhua Ju

**Affiliations:** 1 Guangzhou Municipal and Guangdong Provincial Key Laboratory of Molecular Target and Clinical Pharmacology, The NMPA and State Key Laboratory of Respiratory Disease, School of Pharmaceutical Sciences and the Fifth Affiliated Hospital, Guangzhou Medical University, Guangzhou 511436, China; 2 CAS Key Laboratory of Tropical Marine Bio-Resources and Ecology, Guangdong Key Laboratory of Marine Materia Medica, RNAM Center for Marine Microbiology, South China Sea Institute of Oceanology, Chinese Academy of Sciences, Guangzhou 510301, China; 3 School of Pharmaceutical Sciences, Shandong University, Jinan 250100, China

**Keywords:** angucycline, glycosylation, biosynthesis, P-1894B, grincamycin, marine *Streptomyces*

## Abstract

Recently, we re-isolated the glycosylated angucycline antibiotics P-1894B (**1**) and grincamycin (**1′**) from the marine-derived *Streptomyces lusitanus* SCSIO LR32 as potent antitumor agents and identified their biosynthesis gene cluster *gcn*. Both P-1894B (**1**) and grincamycin (**1′**) possess a trisaccharide and a disaccharide moiety comprised of five deoxysugars. In this work, three genes encoding glycosyltransferases (GcnG1, GcnG2, and GcnG3) responsible for the assembly of deoxysugars into angucycline aglycone were identified from the biosynthesis gene cluster *gcn*. Gene inactivations of *gcnG1*, *gcnG2*, *gcnG3*, and *gcnG1G2* by lambda-RED-mediated gene replacements led to the construction of four mutants, in which the glycosyltransferase genes were disrupted, respectively. The metabolites from the mutants were purified and identified, including two new analogues designated as grincamycin U (**3a**) and V (**3′**). The sequential glycosylation steps in the biosynthesis of P-1894B (**1**) and grincamycin (**1′**) catalyzed by GcnG3, GcnG1, and GcnG2 were elucidated.

## 1. Introduction

Natural angucycline compounds were a group of secondary metabolites derived from *Actinomycetes*, mainly from the genus of *Streptomyces* [1]. Biosynthetically, the initial framework of angucyclines, benz[a]anthracene, was synthesized by type II polyketide synthases (PKS) as angular decaketide intermediate. The benz[a]anthracene backbone was modified by various post-PKS tailoring enzymes to form a multiplicity of sugarless angucycline species, including classical tetracyclic angucycline aglycones with or without angular oxygen atom(s), non-classical tricyclic or rearranged “linear” angucycline aglycones [2]. Most of the angucycline compounds were glycosylated by deoxysaccharide(s), such as D-olivose, L-rhodinose, L-aculose, L-cinerulose A/B, and so on. The glycodiversity leads to dramatic structural diversity of angucyclines. In addition, glycosylated modifications of angucycline aglycones played important roles in enhancing the biological activities of these compounds through improving water solubility and increasing cell membrane-penetrating ability [2]. A typical example was landomycins A and Y bearing a hexasaccharidal chain showed better cytotoxicities against breast cancer cell lines MCF-7 and MDA-231 than other landomycins with shorter sugar chains [3]. The representative angucyclines with significant anti-tumor bioactivities, including BE-7585A, benzanthrin A, grincamycin, vineomycin B2, and kerriamycins A–C, were all glycosylated with deoxysaccharides. The glycosylation of natural angucyclines was catalyzed by glycosyltransferases (GTs) via transferring NDP-activated sugars to the angucycline acceptors [4]. Up to now, dozens of GTs that participated in the assembly of deoxysaccharide chains for landomycin A [5], urdamycin A [6,7], squayamycin [8,9], mithraycin [10], Sch47554, and Sch47555 [11] had been reported. These GTs involved in biosynthesis of angucyclines were divided into the C-GT group and the O-GT group, governing the construction of C-glycoside and O-glycoside via the formation of the C-C bond and C-O bond, respectively. In some cases, one GT could assemble two sugar units through iterative action, such as SaqGT1, SaqGT3, LanGT1, and LanGT4. In the previous reports, the functions of these GTs were elucidated by gene inactivation and heterologous expression experiments. During these courses, some new “unnatural” natural angucycline compounds, such as novel glycosylated urdamycin and landomycin, were produced by genetically constructed mutants, which provided an alternative route to obtain new chemical structures of angucycline by avoiding complicated synthesis reactions of multichiral center deoxysugars.

**Figure 1:**
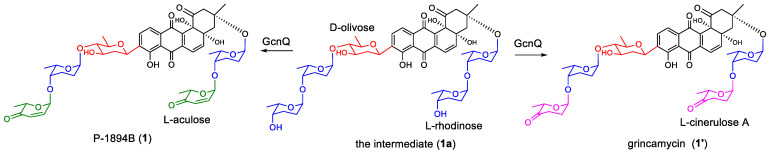
The structures of P-1894B (**1**), grincamycin (**1′**), and the intermediate (**1a**).

The potent antitumor antibiotics P-1894B (**1**, also named vinoemycin A1, Figure 1) [12,13,14] and grincamycin (**1′**) [15,16] were classic angucycline glycosides first identified from the genus of *Streptomyces* in the 1980s. P-1894B (**1**) showed powerful growth inhibition activity toward Jurkat T cells with an IC_50_ value of 11 nmol/L [17]. P-1894B (**1**) also demonstrated in vivo activity against Sarcoma 180 solid tumors in mice. Grincamycin (**1′**) was reported to inhibit the proliferation of murine leukemia P388 cells with an IC_50_ value of 13 ng/mL [15]. The structures of P-1894B (**1**) and grincamycin (**1′**) were very similar; both of them had an identical angucycline aglycone designated SS-228Y-type [1]. P-1894B (**1**) carried a trisaccharide chain comprised of a D-olivose, an L-rhodinose, and an L-aculose at the C-9 position and a disaccharide chain including an L-rhodinose and an L-aculose at the C-3 position. However, the two terminal L-aculose units were replaced by L-cinerulose A units in grincamycin (**1′**). Although the biosynthesis pathway of angucycline aglycone had been investigated [18], the glycosylation pathway of the trisaccharide and disaccharide chains in P-1894B (**1**) and grincamycin (**1′**) was not clear.

In our previous work, we re-isolated P-1894B (**1**) and grincamycin (**1′**) as predominant products from a deep-sea-derived *Streptomyces lusitanus* SCSIO LR32 when fermented in modified AM2 and RA media, respectively [16,17]. The gene cluster *gcn* (NCBI accession number KC962511) responsible for biosynthesis of P-1894B (**1**) and grincamycin (**1′**) was also identified [19]. The *gcn* cluster encoded type II polyketide synthases and cyclases (GcnHIJKL), accounting for angucycline core construction, oxidoreductases (GcnACFMQTU) required for postmodifications, as well as eight deoxysugar biosynthesis enzymes (GcnS1–S8) and three putative glycosyltransferases (GTs, GcnG1–G3). We also uncovered the role of the sugar-tailoring enzyme GcnQ. A glycosidic intermediate (**1a**, Figure 1) with two terminal L-rhodinoses in the di- and trisaccharide chains was obtained from the mutant *Streptomyces lusitanus* SCSIO LR32/Δ*gcnQ*. The two terminal L-rhodinose units in the intermediate (**1a**) were converted into L-aculose units to form P-1894B, or converted into L-cinerulose A to produce grincamycin under the catalyzation of GcnQ, respectively [19]. However, the assembly steps of the D-olivose and four L-rhodinose units in the intermediate (**1a**) were still unknown. In this paper, the role of the three GTs encoded by *gcnG1*~*G3* were investigated by the gene inactivation approach, and the biosynthetic pathway for glycosylation of P-1894B and grincamycin was elucidated. Furthermore, two new angucycline glycosides were obtained from the GT mutant strains, demonstrating that inactivation of GT genes is a promising route to generate a variety of “unnatural” natural compounds.

## 2. Results

The biosynthetic gene cluster *gcn* contains three putative glycosyltransferase genes, including *gcnG1*, *gcnG2*, and *gcnG3*. The BLAST analysis of the primary amino acid sequence of these genes revealed that *gcnG1* and *gcnG2* were homologies to O-GTs, whereas *gcnG3* was homology to C-GTs (Table S1, Supplementary Materials). A comparison of multiple amino acid sequences revealed that GcnG1 showed homology to SaqGT2 with 54% identity [8] and to LanGT4 with 45% identity [20]. The GcnG2 was homologous to SaqGT4 and UrdGT1c with 58% identity [6,7,8]. The GcnG3 showed similarity to UrdGT2 with 79% identity and to SaqGT5 with 65% identity [6,7,8]. Multiple alignments of amino acid sequences inferred that the GcnG1 and GcnG2 may play a role in governing the transfer of the L-rhodinose unit, whereas the GcnG3 could be responsible for the attachment of D-olivose moiety in the biosynthesis of P-1894B (**1**) and grincamycin (**1′**). To confirm the functions of GcnG1, GcnG2, and GcnG3, four mutant strains named *Streptomyces lusitanus* SCSIO LR32/Δ*gcnG1*, Δ*gcnG2*, Δ*gcnG3*, and Δ*gcnG1G2* were constructed by replacing the abovementioned genes using the lambda-RED-mediated gene recombineering strategy [19]. The mutants were fermented in modified AM2 medium. Subsequently, the culture extracts were analyzed by LC–MS; the resultant compounds were isolated by silica gel column chromatography and semi-preparative HPLC. The structures were identified by HR-MS and NMR methods.

### 2.1. Construction and Metabolite Analysis of Mutant ΔgcnG3

The *gcnG3* gene was inactivated using homologous recombination by replacing a 687 bp fragment in *gcnG3* with apramycin resistance gene cassettes *aac(3)IV-oriT* in a size of 1369 bp, which contained the apramycin-resistant gene and the origin of transfer site, to generate a mutant Δ*gcnG3* (Figure S1(A1)). PCR analysis using genome-specific primers (G3tF and G3tR, Table S3) showed a product of 1.7 kb amplified from *Streptomyces lusitanus* SCSIO LR32/Δ*gcnG3* genome, whereas the wild-type genome demonstrated a fragment of 1.0 kb (Figure S1(B1)). These results confirmed the occurrence of the expected double crossover events and verified the inactivation of the *gcnG3* gene.

**Figure 2:**
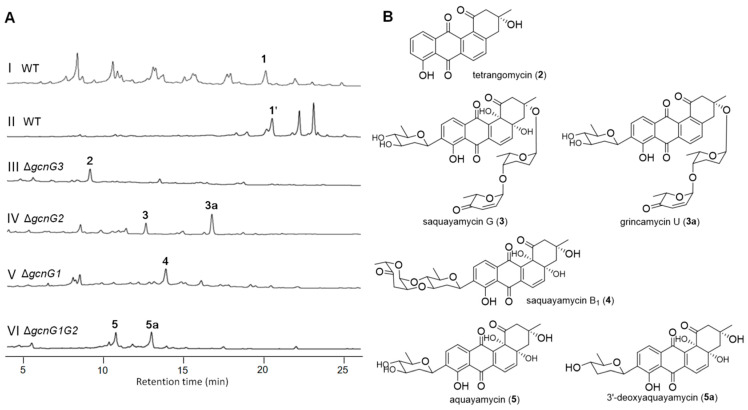
(**A**) HPLC analysis results of the culture products (λ = 254 nm): (I) SCSIO LR32 WT strain fermented in modified AM2 medium; (II) SCSIO LR32 WT strain fermented in modified RA medium; (III)~(VI) Δ*gcnG3*, Δ*gcnG2*, Δ*gcnG1*, and Δ*gcnG1G2* in modified AM2 medium, respectively. (**B**) Compounds isolated from the four mutants fermented in modified AM2 medium.

The mutant Δ*gcnG3* was fermented in modified AM2 medium on a rotation at 28 °C and 200 rpm for 5.5 days in an 8 L scale. After fermentation, the whole culture was centrifuged, and the mycelium was extracted by acetone. LC–UV/MS analysis of the extract showed a predominant peak (**2**) at 8.9 min (Figure 2A, trace III). ESI-MS spectrum revealed a molecular weight of 322 for **2**, suggesting that **2** was an aglycone of angucycline without glycosylation. Subsequently, compound **2** was purified using silica gel column chromatography and semi-preparative HPLC equipped with an ODS column. Comparison of the ^1^H and ^13^C NMR data of **2** with those reported in the literature confirmed that compound **2** was tetrangomycin (Figure 2B) [21].

### 2.2. Construction and Metabolite Analysis of Mutants ΔgcnG2 and ΔgcnG1

The gene *gcnG2* encodes a putative O-glycosyltransferase. The mutant Δ*gcnG2* was constructed using homologous recombination by substituting a 717 bp fragment in *gcnG2* with apramycin-resistant gene cassettes *aac(3)IV-oriT*, as abovementioned (Figure S1(A2)). PCR product amplified from *Streptomyces lusitanus* SCSIO LR32/Δ*gcnG1* genome using genome-specific primers (G2tF and G2tR, Table S3) showed a band with the size of 1.7 kb when analyzed by gel electrophoresis, whereas the wild-type genome displayed a fragment of 1.0 kb (Figure S1(B2)), confirming the generation of the double crossover events.

**Figure 3:**
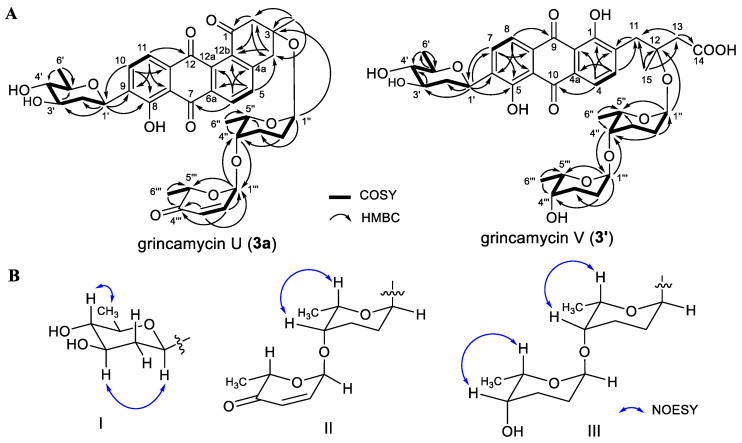
(**A**) The COSY and HMBC correlations of grincamycins U (**3a**) and V (**3′**). (**B**) The key NOESY correlations of the deoxysugar units in grincamycins U (**3a**) and V (**3′**).

The mutant Δ*gcnG2* was fermented and the extract was analyzed in the same fashions as mentioned for Δ*gcnG3*. Two major product peaks with retention times of 12.3 min (**3**) and 16.5 min (**3a**) were revealed in the HPLC–UV/MS chromatography (Figure 2A, trace IV). Compound **3** was identified to be saquayamycin G by comparing its ^1^H and ^13^C NMR data with those reported in previous literature [22]. HR-ESI-MS spectrum of **3a** showed an [M−H]^−^ ion signal at *m*/*z* 675.2448, suggestive of a molecular formula of C_37_H_40_O_12_. The ^1^H and ^13^C NMR spectroscopic data of **3a** were similar to those of saquayamycin G (Table 1). Detailed comparison revealed that two oxygen-bearing quaternary carbons at *δ*_C_ 81.1 (C-4a) and *δ*_C_ 83.4 (C-12b) in saquanyamycin G were replaced by two aromatic carbons at *δ*_C_ 149.7 (C-4a) and *δ*_C_ 136.7 (C-12b) in **3a**, respectively. The HMBC correlations from H-5 (*δ*_H_ 7.56) to C-4, C-6a, and C-12b, and from H-6 (*δ*_H_ 8.06) to C-4a, C-7, and C-12a, confirmed the presence of a benzene ring. Therefore, compound **3a** was determined to be a new analog of saquanyamycin G, and only the aglycone was replaced by tetrangomycin. Analysis of 2D NMR (COSY, HMQC, HMBC, and NOESY) spectroscopic data of **3a** (Figure 3) verified the elucidated structure. Compound **3a** was named grincamycin U.

The *gcnG1* is homologous to O-GTs. The Δ*gcnG1* mutant strain was constructed and fermented by the same approach. HPLC–UV/MS analysis of culture extract of the mutant Δ*gcnG1* revealed a major product with a retention time of 13.8 min (**4**) (Figure 2A, trace V). Compound **4** was purified for structure identification. A comparison of the ^1^H and ^13^C NMR data of **4** with those previously reported in the literature indicated that **4** was saquayamycin B_1_ [22,23].

### 2.3. Construction and Metabolite Analysis of Mutant ΔgcnG1G2

The two genes *gcnG1* and *gcnG2* were next to each other closely in the alignment of the *gcn* gene cluster. Therefore, a mutant with double deletion of *gcnG1* and *gcnG2* was constructed successfully using the forward primer of *gcnG1* and the reverse primer of *gcnG2* by replacing a fragment in *gcnG1* and *gcnG2* with *aac(3)IV-oriT* cassettes (Figure S1(A4)). After verification by gel electrophoresis analysis of PCR products amplified from *Streptomyces lusitanus* SCSIO LR32/Δ*gcnG1G2* genome (Figure S1(B4)), the mutant Δ*gcnG1G2* was fermented and extracted using the same fashions as mentioned above. HPLC–UV/MS analysis revealed two product peaks with retention times of 10.7 min (**5**) and 12.9 min (**5a**) (Figure 2A, trace VI), demonstrating molecular weights of 486 and 470, respectively. Compounds **5** and **5a** were purified in a quantity sufficient for measurement of ^1^H and ^13^C NMR. These two compounds were determined to be aquayamycin (**5**) [24,25] and 3′-deoxyaquayamycin (**5a**) [11] by comparison of the ^1^H and ^13^C NMR data with those previously reported, respectively.

### 2.4. Products of Mutant ΔgcnG3~ΔgcnG1 and ΔgcnG1G2 in Modified RA Medium

**Figure 4:**
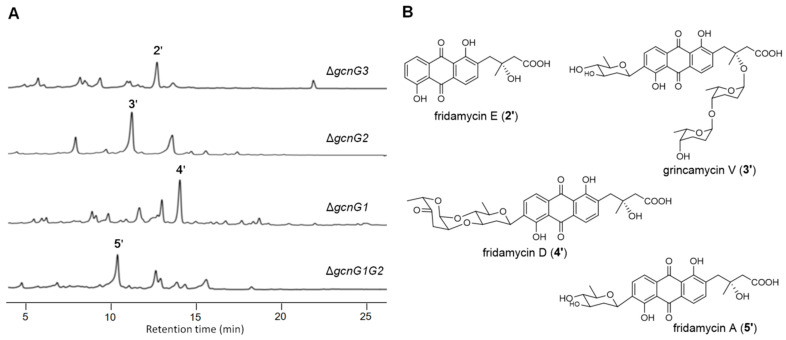
(**A**) HPLC profile (λ = 254 nm) of the four mutants cultured in modified RA medium. (**B**) Chemical structures of compounds isolated from the mutants cultured in modified RA medium.

It is interesting to note that when the SCSIO LR32 wild-type strain fermented in modified RA medium, several nonclassic angucycline C-glycosides with rearranged tricyclic aglycone (fridamycin E, 2′) were obtained, including grincamycins B, C, D, and K [16,26]. In order to confirm if the mutants fermented in modified RA medium could produce compounds with the same tricyclic aglycone, all four mutants were fermented again using modified RA medium. Not surprisingly, the isolation and identification of the culture products revealed the presence of the tricyclic fridamycin E (2′) [27] from Δ*gcnG3*. Additionally, four C-glycosides carried the fridamycin E (2′) as aglycone were obtained, including a new compound named grincamycin V (3′) from Δ*gcnG2*, fridamycin D (4′) [28] from Δ*gcnG1*, and fridamycin A (5′) [29] from Δ*gcnG1G2* (Figure 4).

The new structure of grincamycin V (**3**′) was determined by the interpretation of MS and NMR data. The HR-ESI-MS signal of **3**′ at *m/z* 713.2796 ([M−H]^−^, calc. for 713.2815, C_37_H_45_O_14_^−^) indicated the molecular formula of C_37_H_46_O_14_. The ^1^H and ^13^C NMR spectroscopic data of **3**′ revealed a C-glycosidic moiety the same as fridamycin A (**5**′). Additional signals attributed to two anomeric methines at *δ*_H_ 5.23 (brs), *δ*_C_ 91.3 (CH-1″) and *δ*_H_ 4.77 (brs), *δ*_C_ 98.6 (CH-1‴) suggested the presence of two more sugar units. The ^1^H−^1^H spin systems revealed in the COSY spectrum and the HMBC correlations established two rhodinose sugars (Figure 3). The O-glycosidic linkages of C-1‴−O−C-4″ and C-1″−O−C-12 were confirmed by the HMBC correlations of H-1‴/C-4″ and H-4″/C-1‴, and H-1″/C-12, respectively.

## 3. Discussion

### 3.1. Functional Characterizations of GcnG1~GcnG3

**Figure 5:**
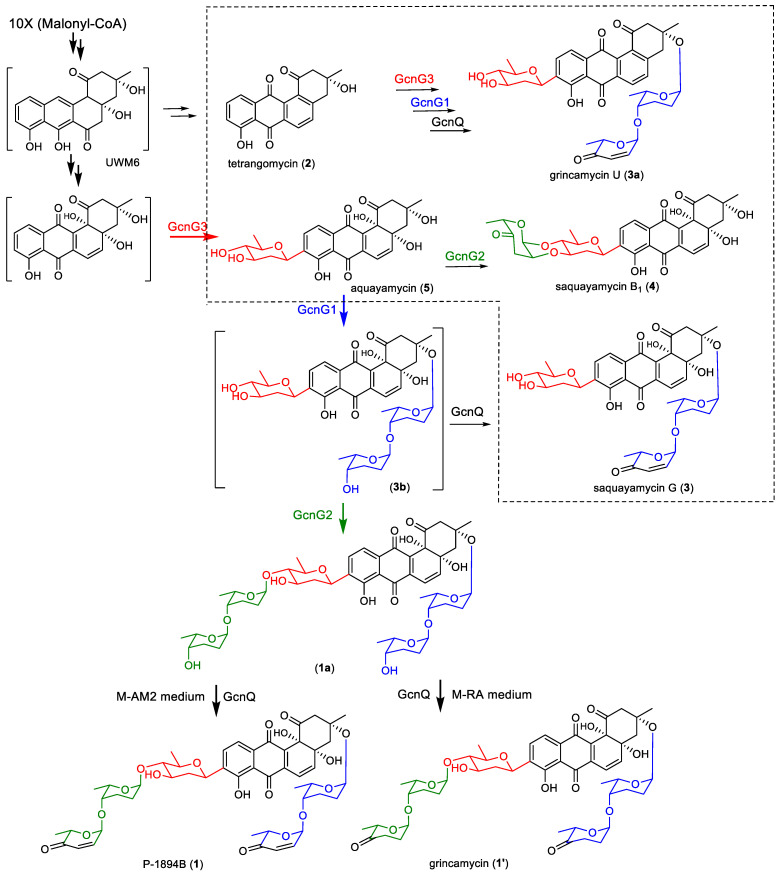
The glycosylation steps catalyzed by GcnG1~GcnG3 (arrows in color) in the biosynthetic pathway of P-1894B (**1**) and grincamycin (**1′**). Compounds in the dotted line frame were isolated from the mutants cultured in modified AM2 medium.

The mutant Δ*gcnG3* produced the aglycone tetrangomycin (**2**) without any sugar. The mutant Δ*gcnG1G2* yielded the C-glycosidic compound aquayamyicn (**5**), and 3′-deoxyaquayamycin (**5a**) only carried a D-olivose and a 3′-deoxy-olivose unit, respectively. These results unambiguously confirmed that the *gcnG3* gene encoded C-glycosyltransferase responsible for the attachment of the D-olivose to form the initial C-glycosidic aquayamyicn (**5**) in the glycosylation pathway. The mutant Δ*gcnG2* generated saquayamycin G (**3**) and grincamycin U (**3a**). In addition to the D-olivose transferred by GcnG3 attached at the C-9 position, compounds **3** and **3a** also carried a disaccharidic chain of L-aculose-(1→4)-L-rhodinose linked at the C-3 position of the aglycone, which was the same as the disaccharide chain in P-1894B (**1**). As we know that the terminal L-aculose in P-1894B (**1**) was converted from an L-rhodinose under the catalyzation of GcnQ [19], compounds **3** and **3a** isolated from Δ*gcnG2* mutant thus suggested that the GcnG1 transferred an L-rhodinose-(1→4)-L-rhodinose moiety into the C-3 position of the aglycone. On the other hand, compounds **3** and **3a** lost the L-aculose-(1→4)-L-rhodinose chain connected with D-olivose relative to P-1894B (**1**), conversely indicating the *gcnG2* gene encoding an O-glycosyltransferase responsible for assembly of the L-rhodinose-(1→4)-L-rhodinose moiety attached in the D-olivose of the intermediate (**1a**) in the wild-type SCSIO LR32 strain. The mutant Δ*gcnG1* produced saquayamycin B_1_ (**4**) bearing an L-cinerulose B-(1→4, 2→3)-D-olivose unit, which only had one more L-cinerulose B unit instead of the expected rhodinose-(1→4)-L-rhodinose. It was speculated that the inherent substrate of GcnG2 in the wild-type SCSIO LR32 strain could be the intermediate (**3b,** Figure 5), which did not exist in the mutant Δ*gcnG1*. Alternatively, GcnG2, a protein with wide substrate specificity, catalyzed a monoglycosylation step using the non-inherent substrate aquayamycin (**5**) to produce a shunt metabolite saquayamycin B_1_ (**4**) in the Δ*gcnG1*. This result suggested that the assembly of the right L-rhodinose-(1→4)-L-rhodinose moiety at C-3 of the aglycone transferred by GcnG1 was prior to the left one linked with D-olivose assembled by GcnG2. Additionally, the absence of the right L-rhodinose-(1→4)-L-rhodinose unit at C-3 in saquayamycin B_1_ (**4**) produced from Δ*gcnG1* conversely confirmed that the diglycosylation step of the L-rhodinose-(1→4)-L-rhodinose was catalyzed by GcnG1.

### 3.2. The Glycosylation Pathway of P-1894B and Grincamycin

The glycosylation step in the biosynthetic pathway of P-1894B (**1**) and grincamycin (**1′**) in the wild-type SCSIO LR32 strain are shown in Figure 5. The aglycone was sequentially glycosylated. Firstly, the C-GT GcnG3 transferred a D-olivose into the C-9 position to form a C-glycosidic aquayamyicn (**5**). Secondly, an L-rhodinose-(1→4)-L-rhodinose chain was attached at C-3 by the O-GT GcnG1, generating the intermediate (**3b**). Then, another L-rhodinose-(1→4)-L-rhodinose chain was assembled onto D-olivose by the O-GT GcnG2 to yield the final intermediate (**1a**). Finally, the two terminal L-rhodinosyl units in **1a** were converted into L-aculoses to produce P-1894B (**1**) or were changed into L-cinerulose A units to form grincamycin (**1′**) under the catalyzation of the oxidoreductase GcnQ in modified AM2 or RA media, respectively. 

### 3.3. The Glycosylation Pathway of Compounds with Tricyclic Aglycone

**Figure 6:**
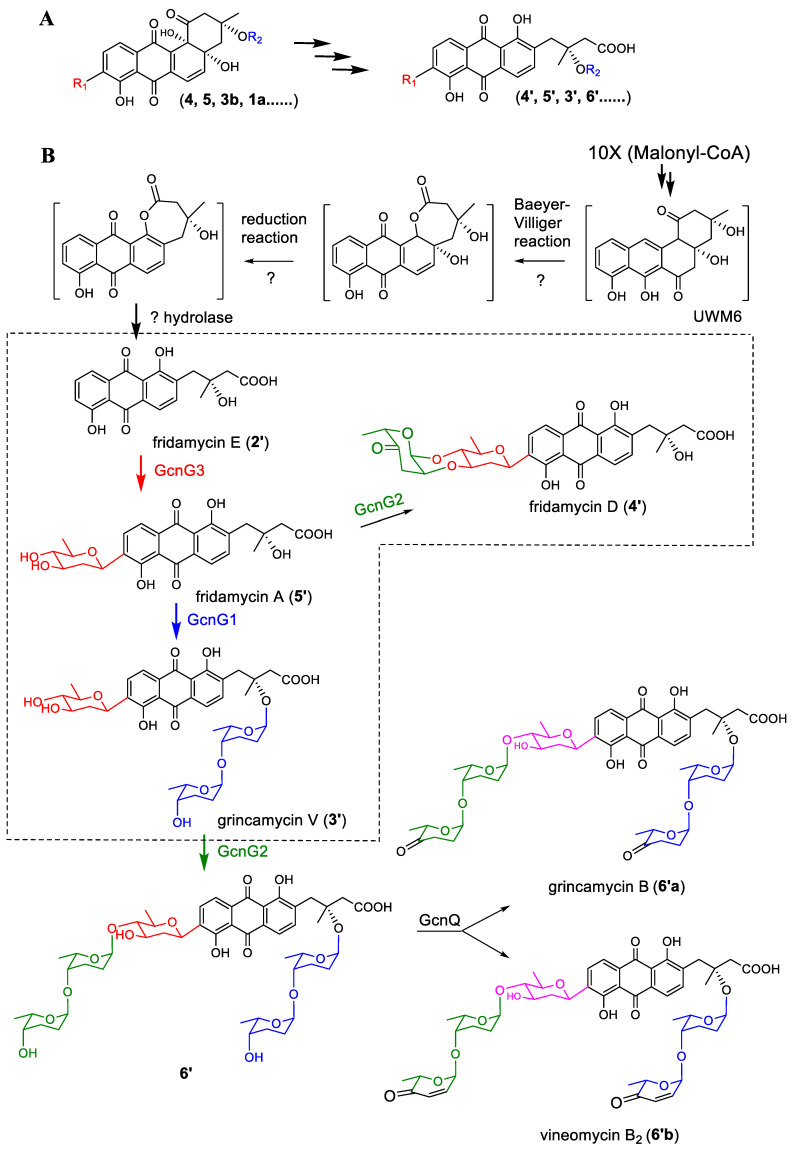
The possible biosynthetic pathways of tricyclic aglycone glycosides. Pathway (**A**) the tetracyclic glycosides were converted into the tricyclic glycosides by biosynthetic enzymes. Pathway (**B**) the tricyclic aglycone fridamycin E (**2′**) was generated first, followed by sequential glycosylation under catalyzation of GcnG3, GcnG1, and GcnG2. Compounds in the dotted line frame were isolated from the mutants fermented in modified RA medium.

The tricyclic fridamycin E (**2′**) and three anguacycline glycosides (**3′**, **4′**, and **5′**) with fridamycin E (**2′**) as aglycone were isolated from the mutants cultured in modified RA medium. Previous reports showed that the classic tetracyclic aglycone could be rearranged into the tricyclic aglycone under acidic conditions [1,19,30]. However, the fermentation, extract, and purification procedures for these tricyclic compounds were not treated with any acids. The O-glycosidic bonds in the structures of grincamycin V (**3′**) and fridamycin D (**4′**) also proved the acid-free isolation process. Thus, the generation of the tricyclic aglycone angucycline glycosides in the mutant and wild-type strains of SCSIO LR32 could be catalyzed by biosynthetic enzymes. One possible biosynthetic pathway could be that the tetracyclic glycosides (**4**, **5**, **3b**, and **1a**) were transferred into the compounds with tricyclic aglycone directly (Figure 6A), while another possibility involves the formation of tricyclic aglycone compounds that may be the common intermediate UWM6 of angucycline converted into fridamycin E (**2′**) firstly, then a serial of glycosylated reactions would occur sequentially under the catalyzation of GcnG3, GcnG1, and GcnG2 to produce the intermediate (**6′**). Compound **6′** was finally transformed into grincamycin B (**6′a**) and vineomycin B2 (**6′b**) as minor products catalyzed by GcnQ in the wild-type strain (Figure 6B).

Our previous works showed that the tricyclic angucycline grincamycin B (**1″**) showed significant inhibitory effects on human acute promyelocytic leukemia NB4 cells and glioblastoma multiforme cells [31,32]. Most recently, several tricyclic aglycone-containing compounds, grincamycins P-T, were identified from a marine-sediment-derived *Streptomyces* stain, which exhibited low micromolar inhibitory activities against pseudomyxoma peritonei cells [33]. However, the detailed biosynthetic pathway for the tricyclic anguacycline products remains to be investigated.

## 4. Materials and Methods

### 4.1. Materials and General Experimental Procedures

The wild-type strain *Streptomyces lusitanus* SCSIO LR32 was isolated from deep-sea-derived sediment collected in the South China Sea in 2010 by our group [16]. Genomic library construction and screening, and DNA sequencing and annotation, had been reported in our previous work [19]. Strains and plasmids used and generated in this study are listed in Table S2 (Supplementary Materials). All DNA manipulations were performed according to standard procedures or the manufacturer’s instructions. Reagents for polymerase chain reactions (PCR), restriction enzymes, and DNA ligase were purchased from Takara Co., Ltd. (Dalian, China) and Trans gene Co., Ltd. (Beijing, China). Primers used for gene disruption and PCR confirmation were synthesized by Sangon Biotech Co., Ltd., (Shanghai, China). DNA sequencing was performed at the Invitrogen Biotech Co., Ltd. (Guangzhou, China), the Major Biotech Co., Ltd. (Shanghai, China), and Chinese National Genome Center (Shanghai, China). Plasmid and gel extraction kits were purchased from Omega Bioteck Inc. (Guangzhou, China). Other chemical and biochemical reagents were purchased from standard commercial sources.

Optical rotations were measured on an MCP 500 polarimeter (Anton Paar, Graz, Austria). The 1D and 2D NMR spectra were recorded with an AV-500 spectrometer (Bruker, Billerica, MA, USA). The chemical shifts were given in ppm relative to the signals from residual undeuterated solvent or TMS. LC–MS analyses with low- and high-resolution mass spectrometry were conducted on a 1260 HPLC instrument (Agilent, Santa Clara, CA, USA) coupled with an Amazon SL ion trap mass spectrometer and a Maxis quadrupole time-of-flight mass spectrometer (Bruker, Billerica, MA, USA) using Luna C18 columns (150 × 4.6 mm, 5 μm, Phenomenex, Torrance, CA, USA), respectively. The elution system consisted of solvent A (15% acetonitrile in H_2_O added with 0.1% HOAc) and solvent B (85% acetonitrile in H_2_O added with 0.1% HOAc). A linear gradient elution started from 20% solvent B to 100% solvent B in 0–20 min was employed, then kept 100% solvent B for 12 min. The flow rate was set at 1 mL/min. Column chromatography (CC) was performed using silica gel (100–200 mesh; Jiangyou Silica gel development, Inc., Yantai, China). Semi-preparative HPLC was performed on a ProStar apparatus (Varian, Palo Alto, CA, USA) using an Ultimate XB-C18 column (250 × 10 mm, 5 µm, Welch, Shanghai, China).

### 4.2. Gene Inactivation Experiments

The gene inactivation experiments in *S. lusitanus* SCSIO LR32 were performed using the lambda-RED-mediated gene replacements as previously reported [19]. In brief, the apramycin resistance gene cassette was amplified using the primers listed in Table S3 and followed by gel purification. The resulting *oriT-aac(3)*IV cassette was introduced into *E. coli* BW25113/pIJ790 containing cosmid 228A by electroporation. Those positive clones in which the targeted gene was replaced by *oriT*-*aac(3)*IV were selected through apramycin resistance. The correctly mutated cosmids were then introduced into *E. coli* ET12567/pUZ8002 for conjugation with *S. lusitanus SCSIO LR32.* The donor *E. coli* ET12567/pUZ8002 was incubated to an OD_600_ value of 0.6~0.8, and then the cells were resuspended in LB medium. Spores of *S. lusitanus* SCSIO LR32 were germinated in TSB medium for 10~12 h and then resuspended in LB medium. Next, 200 μL of donor strains and 200 μL of recipient strains were mixed and spread onto M-ISP4 (soluble starch 1%, K_2_HPO_4_ 0.1%, MgSO_4_·7H_2_O 0.1%, NaCl 0.1%, (NH_4_)_2_SO_4_ 0.2%, CaCO_3_ 0.2%, peptone 0.1%, yeast extract powder 0.05%, trace salt 0.1 mL, sea salt 3%, pH 7.0~7.4) medium plates and grown for 20 h at 30 °C. The plates were covered with 25 μL apramycin (50 mg/mL) and 25 μL trimethoprim (50 mg/mL) and then incubated at 30 °C after drying. The exconjugants with apramycin-resistant characteristics were selected 4~6 d later. The double-crossover mutants from homologous recombination were verified through diagnostic PCR using primers listed in Supplementary Table S4. The PCR confirmation of the mutants is shown in Figure S1(B1~B4). Three independent clones from each double-crossover mutant were randomly chosen for small-scale fermentation and subjected to metabolite analyses by HPLC. In each case, three independent clones showed the same profile of metabolites, providing reliable evidence for the inactivation of the target genes. In total, four genetic mutants *S. lusitanus* SCSIO LR32/Δ*gcnG1*, Δ*gcnG2*, Δ*gcnG3*, and Δ*gcnG1G2* were successfully constructed.

### 4.3. Fermentation of Mutant Strains

The fermentation method adopted for the *S. lusitanus* SCSIO LR32 wild-type strain and mutant strains has been reported previously [19]. The spore of wild-type or mutant strains was seeded into 50 mL of modified AM2 medium in a 250 mL flask and incubated on a rotary shaker under 200 rpm at 28 °C for 1.5 days. Then, 10% (*v/v*) seed culture was transferred into 200 mL modified AM2 medium or modified RA medium in a 1 L flask and then incubated on a rotary shaker under 200 rpm at 28 °C for 5 days.

Modified AM2 medium: bean powder 0.5%, yeast extract 0.2%, peptone 0.2%, soluble starch 0.5%, glucose 2%, CaCO_3_ 0.2%, NaCl 0.4%, KH_2_PO_4_ 0.05%, MgSO_4_ 0.05%, sea salt 3%, pH 7.2~7.4 before sterilization.

Modified RA medium: glucose 1%, malt extract 1%, corn starch 0.5%, soluble starch 2%, maltose 1%, trace elements (100 mL/L), sea salt 3%, pH 7.2~7.4 before sterilization.

### 4.4. Isolation of Grincamycin Analogues from Mutant Strains

When the fermentation was finished, the whole culture broth (8 L for each strain) was centrifuged to yield mycelium and supernatant. The mycelium was extracted with an equal volume of acetone to afford an oily residue. The residue was subjected to silica gel CC using gradient elution with mixtures of CHCl_3_/MeOH (100:0, 99:1, 97:3, 95:5, 93:7, 90:10, *v/v*) to give six fractions (Fr.1–Fr.6). Then, the fractions containing targeted compounds were further separated by silica gel CC eluted with petroleum ether (60~90 °C)/EtOAc or by preparative HPLC eluted with CH_3_CN-H_2_O. For example, Fr.2 from the mutant Δ*gcnG3* was isolated by silica gel CC eluted with mixtures of petroleum ether (60~90 °C)/EtOAc (80:20, 70:30, 60:40, 50:50, 40:60, 30:70, 20:80, 10:90, *v*/*v*) to afford eight fractions (Fr.2-1~Fr.2-8). Fr.2-6 was purified by semi-preparative HPLC equipped with an Ultimate C18 column (250 × 21.2 mm, 5 μm), eluting with 30% HCN to 70% HCN over 30 min at a flow rate of 2.5 mL/min to yield 3′-deoxyaquayamycin (**5a**, t_R_ 18.2 min). Fr. 3 was isolated by silica gel CC under the same elution method to give eight fractions (Fr.3-1~Fr.3-8). Fr.3-6 and Fr.3-7 were combined and purified by semi-preparative HPLC, eluting with 30% HCN to 90% HCN over 35 min at a flow rate of 2.5 mL/min to yield aquayamycin (**5**, t_R_ 15.3 min). Detailed isolation processes were introduced in Figures S2–S5 (Supplementary Materials).

Grincamycin U (**3a**): dark-red powder; [α]^20^_D_ + 165 (*c* 0.20, MeOH); UV (MeOH) *λ*_max_ (log *ε*) 230 (4.39), 258 (4.15), 294 (3.68), 442 (3.79); ^1^H NMR and ^13^C NMR data, see Table 1; (−)HR-ESI-MS *m/z* 675.2448 ([M−H]^−^, calcd for C_37_H_39_O_12_^−^, 675.2447).

Grincamycin V (**3′**): dark-red powder; [α]^20^_D_ + 231 (*c* 0.20, MeOH); UV (MeOH) *λ*_max_ (log *ε*) 232 (4.42), 259 (4.17), 293 (3.67), 446 (3.74); ^1^H NMR and ^13^C NMR data, see Table 1; (−)HR-ESI-MS *m/z* 713.2796 ([M−H]^−^, calcd for C_37_H_45_O_14_^−^, 713.2815).

## 5. Conclusions

In sum, four mutants with disruption of glycosyltransferase genes (*gcnG1*, *gcnG2*, *gcnG3*, and *gcnG1G2*) were constructed by the lambda-RED-mediated gene replacement approach on the basis of identification of the biosynthetic gene cluster for P-1894B (**1**) and grincamycin (**1′**). The angucycline analogs from the mutants were purified and identified, including two new glycosidic compounds designated grincamycins U (**3a**) and V (**3′**). The functions of the three glycosyltransferases GcnG1, GcnG2, and GcnG3 were characterized, and the glycosylation steps in the biosynthetic pathway of P-1894B (**1**) and grincamycin (**1′**) catalyzed by these glycosyltransferases were elucidated based on the analyses of chemical structures obtained from the mutants. In our future endeavors, we aim to diversify angucycline glycoside compounds by integrating these glycosyltransferase genes into other related biosynthetic gene clusters.

## Figures and Tables

**Table 1:** ^1^H (700 MHz) and ^13^C (175 MHz) NMR data for gincamycins U (**3a**) and V (**3′**) in CD_3_OD.

	Gincamycin U (3a)			Gincamycin V (3′)	
	*δ*_C_, Type	*δ*_H_, multi. (*J* in Hz)		*δ*_C_, Type	*δ*_H_, multi. (*J* in Hz)
**1**	198.6, C		**1**	160.9, C	
**2**	52.8, CH_2_	3.18, d (15.5);	**2**	134.9, C	
		2.87, d (15.5)			
**3**	77.8, C		**3**	139.5, CH	7.60, d (7.7)
**4**	42.6, CH_2_	3.29, d (16.5);	**4**	118.7, CH	7.42, d (7.7)
		3.10, d (16.5)			
**4a**	149.7, C		**4a**	131.2, C	
**5**	134.4, CH	7.56, d (7.3)	**5**	158.1, C	
**6**	130.3, CH	8.06, d (7.3)	**6**	138.2, C	
**6a**	134.5, C		**7**	132.7, CH	7.70, d (7.7)
**7**	188.7, C		**8**	117.9, CH	7.45, d (7.7)
**7a**	115.8, C		**8a**	131.1, C	
**8**	158.9, C		**9**	187.3, C	
**9**	138.2, C		**9a**	114.9, C	
**10**	134.8, CH	7.75, d (7.7)	**10**	187.3, C	
**11**	119.9, CH	7.42, d (7.7)	**10a**	114.7, C	
**11a**	135.0, C		**11**	38.0, CH_2_	3.21, d (13.0); 3.06, d (13.0)
**12**	183.8, C		**12**	77.2, C	
**12a**	136.7, C		**13**	44.1, CH_2_	2.76, d (15.0); 2.61, d (15.0)
**12b**	136.7, C		**14**	173.3, C	
**13**	26.3,CH_3_	1.49, s	**15**	22.3, CH_3_	1.44, s
olivose			olivose		
**1′**	72.4, CH	4.77, d (11.2)	**1′**	71.1, CH	4.74, d (11.9)
**2′**	40.9, CH_2_	2.46, dd (11.2, 4.0);	**2′**	39.5, CH_2_	2.49, dd (11.9, 3.5);
		1.37, t (11.3)			1.40, m
**3′**	73.6, CH	3.72, ddd (11.4, 9.0, 4.9)	**3′**	72.3, CH	3.74, m
**4′**	78.8, CH	3.06, t (9.0)	**4′**	77.4, CH	3.10, t (8.9)
**5′**	77.7, CH	3.46, m	**5′**	76.3, CH	3.44, m
**6′**	18.8, CH_3_	1.39, d (6.0)	**6′**	17.4, CH_3_	1.42, overlapped
rhodinose			rhodinose 1		
**1″**	92.9, CH	5.10, brs	**1″**	91.3, CH	5.23, brs
**2″**	26.0, CH_2_	1.80, m; 1.50, m	**2″**	25.4, CH_2_	2.08, m; 1.42, m
**3″**	25.2, CH_2_	1.70, m	**3″**	24.3, CH_2_	2.10, m; 1.85, m
**4″**	77.6, CH	3.49, s	**4″**	75.1, CH	3.48, m
**5″**	67.8, CH	3.97, q (6.5)	**5″**	66.7, CH	4.11, m
**6″**	17.5, CH_3_	1.12, d (6.5)	**6″**	16.2, CH_3_	1.10, d (6.4)
aculose			rhodinose 2		
**1** **‴**	96.4, CH	5.19, d (3.2)	**1** **‴**	98.6, CH	4.77, brs
**2** **‴**	145.5, CH	6.95, dd (10.2, 3.2)	**2** **‴**	29.5, CH_2_	1.93, m; 1.69, m
**3** **‴**	127.5, CH	6.00, d (10.2)	**3** **‴**	27.1, CH_2_	1.80, m
**4** **‴**	198.4, C		**4** **‴**	71.4, CH_2_	3.18, m
**5** **‴**	71.4, CH	4.47, q (6.7)	**5** **‴**	70.0, CH	3.69, m
**6** **‴**	15.5, CH_3_	1.17, d (6.7)	**6** **‴**	17.0, CH_3_	1.44, overlapped

## Data Availability

All data supporting the findings of this study are available from the corresponding author upon reasonable request.

## Supplementary Materials

The following supporting information can be downloaded at: https://www.mdpi.com/article/10.3390/md22010032/s1, Figure S1: Gene inactivation of *gcnG3*~*gcnG1*, and *gcnG1G2*; Figures S2–S5: Procedures for isolation of compounds from the mutants; Figures S6–S23: The UV, (−)HR-ESI-MS, and NMR spectra of grincamycin U (**3a**) and grincamycin V (**3′**); Table S1: Deduced functions of ORFs containing *gcnG1*~*gcnG3* and *gcnQ* genes in the grincamycin biosynthetic gene cluster; Tables S2–S4: Bacteria, plasmid, and primers used in this study.
