# Supplementary material for: Deciphering the Glycosylation Steps in the Biosynthesis of P-1894B and Grincamycin Isolated from Marine-Derived Streptomyces lusitanus SCSIO LR32
Source: Mar Drugs. 2024 Jan 2;22(1):32. doi: 10.3390/md22010032 (PMC10817425; doi:10.3390/md22010032)

Supplementary Materials

Deciphering the Glycosylation Steps in the Biosynthesis of P-1894B and Grincamycin Isolated from Marine-derived Streptomyces lusitanus SCSIO LR32.

Hongbo Huang [1,2,†], Yun Zhang [2,†], Yongxiang Song [2], Chunyao Ling [2], Siyan Peng [1], Bo Ding [1], Yiwen Tao [1,*] and Jianhua Ju [2,3,*]

[1] Guangzhou Municipal and Guangdong Provincial Key Laboratory of Molecular Target and Clinical Pharmacology, the NMPA and State Key Laboratory of Respiratory Disease, School of Pharmaceutical Sciences and the Fifth Affiliated Hospital, Guangzhou Medical University, Guangzhou 511436, China; huanghb@gzhmu.edu.cn (H. H.), 18054290097@163.com (S.P.), dingbo-dmy@gzhmu.edu.cn (B.D.)
[2] CAS Key Laboratory of Tropical Marine Bio-resources and Ecology, Guangdong Key Laboratory of Marine Materia Medica, RNAM Center for Marine Microbiology, South China Sea Institute of Oceanology, Chinese Academy of Sciences, Guangzhou 510301, China; zhangyun@scsio.ac.cn (Y.Z.), songx@scsio.ac.cn (Y.S.), lingchunyao071@163.com (C.L.)
[3] School of Pharmaceutical Sciences, Shandong University, Jinan 250100, China
† Huang H. and Zhang Y. contributed equally.
* Correspondence: jju@scsio.ac.cn(J.J.), Tel.: +86-20-89023028 (J.J.); yywentao@aliyun.com(Y.T.), Tel.: +86-20-37103255 (Y.T.)

Abstract: Recently, we re-isolated the glycosylated angucycline antibiotics P-1894B (**1**) and grincamycin (**1'**) from the marine-derived *Streptomyces lusitanus* SCSIO LR32 as potent antitumor agents and identified their biosynthesis gene cluster *gcn*. Both P-1894B (**1**) and grincamycin (**1'**) possess a trisaccharide and a disaccharide moiety comprised of five deoxysugars. In this work, three genes encoding glycosyltransferases (GcnG1, GcnG2, and GcnG3) responsible for the assembly of deoxysugars into angucycline aglycone were identified from the biosynthesis gene cluster *gcn*. Gene inactivations of *gcnG1*, *gcnG2*, *gcnG3*, and *gcnG1G2* by lambda-RED-mediated gene replacements led to the construction of four mutants, in which the glycosyltransferase genes were disrupted, respectively. The metabolites from the mutants were purified and identified, including two new analogues designated as grincamycin U (**3a**) and V (**3'**). The sequential glycosylation steps in the biosynthesis of P-1894B (**1**) and grincamycin (**1'**) catalyzed by GcnG3, GcnG1, and GcnG2 were elucidated.

Keywords: angucycline; glycosylation; biosynthesis; P-1894B; grincamycin; marine *Streptomyces*

Table of Contents

Tables	Content	Page
**Table S1**	Deduced functions of ORFs containing *gcnG1~G3* and *gcnQ* genes in the grincamycin biosynthetic gene cluster.	S3
**Table S2**	Bacteria and plasmid used in this study.	S3
**Table S3**	The primer pairs used for mutant construction.	S4
**Table S4**	The primers used for PCR confirmation of the double-crossover mutants.	S4
**Figures**		
**Figure S1**	Gene inactivation of *gcnG3~gcnG1*, and *gcnG1G2*.	S5
**Figure S2**	Procedure for isolation of compound from the mutant Δ*gcnG3*.	S5
**Figure S3**	Procedure for isolation of compounds from the mutant Δ*gcnG2*.	S6
**Figure S4**	Procedure for isolation of compound from the mutant Δ*gcnG1*.	S6
**Figure S5**	Procedure for isolation of compound from the mutant Δ*gcnG1G2*.	S7
**Figure S6**	The UV spectrum of grincamycin U (**3a**).	S7
**Figure S7**	The (-)HR-ESI-MS spectrum of grincamycin U (**3a**).	S7
**Figure S8**	The ^{1}H NMR (700 MHz) spectrum of grincamycin U (**3a**) in CD_3OD.	S8
**Figure S9**	The ^{13}C NMR (175 MHz) spectrum of grincamycin U (**3a**) in CD_3OD.	S9
**Figure S10**	The DEPT-135 (175 MHz) spectrum of grincamycin U (**3a**) in CD_3OD.	S10
**Figure S11**	The COSY NMR spectrum of grincamycin U (**3a**) in CD_3OD.	S11
**Figure S12**	The HMQC NMR spectrum of grincamycin U (**3a**) in CD_3OD.	S12
**Figure S13**	The HMBC NMR spectrum of grincamycin U (**3a**) in CD_3OD.	S13
**Figure S14**	The NOESY NMR spectrum of grincamycin U (**3a**) in CD_3OD.	S14
**Figure S15**	The UV spectrum of grincamycin V (**3'**).	S15
**Figure S16**	The (-)HR-ESI-MS spectrum of grincamycin V (**3'**).	S15
**Figure S17**	The ^{1}H NMR (700 MHz) spectrum of grincamycin V (**3'**) in CD_3OD.	S16
**Figure S18**	The ^{13}C NMR (175 MHz) spectrum of grincamycin V (**3'**) in CD_3OD.	S17
**Figure S19**	The DEPT-135 (175 MHz) spectrum of grincamycin V (**3'**) in CD_3OD.	S18
**Figure S20**	The COSY NMR spectrum of grincamycin V (**3'**) in CD_3OD.	S19
**Figure S21**	The HMQC NMR spectrum of grincamycin V (**3'**) in CD_3OD.	S20
**Figure S22**	The HMBC NMR spectrum of grincamycin V (**3'**) in CD_3OD.	S21
**Figure S23**	The NOESY NMR spectrum of grincamycin V (**3'**) in CD_3OD.	S22

Table S1. Deduced functions of ORFs containing *gcnG1~G3* and *gcnQ* genes in the grincamycin biosynthetic gene cluster.

gene	Size(a.a)	Proposed function	Protein homologue and origin	Identity/ similarity	accession number
gcnG1	430	O-Glycosyltransferase	SaqGT2[*Micromonospora sp*. Tu 6368]	54/69	ACP19363.1
gcnG2	404	O-Glycosyltransferases	SaqGT4[*Micromonospora sp*. Tu 6368]	58/70	ACP19365.1
gcnG3	376	C-Glycosyltransferase	UrdGT2[*Streptomyces fradiae* Tue2717]	79/85	AAF00209.1
gcnQ	529	FAD/FMN-containin-g dehydrogenases	AknOx[*Streptomyces galilaeus* ATCC31615]	56/69	ABI15166.1

Table S2. Bacteria and plasmid used in this study.

Strains	Description	Reference or source
E. coli		
ET12567	*dam, dcm, hsdS, cat, tet*	[MacNeil, 1992]
BW25113	K12 derivative: *araBAD, rhaBAD*	[Datsenko, 2000]
Plasmids		
pCR2.1	Amp^r, Kan^r, general clone vector	Invitrogen
pIJ790	Cml^r, including λ-RED (*gam, bet, exo*) for PCR-targeting	This study
pIJ773	*aac(3)IV* (ApraR), *oriT*	This study
pUZ8002	*tra, neo, RP4*	[Paget, 1999]
Cosmid		
228A	*Streptomyces lusitanus* SCSIO LR32 genomic library cosmid	This study
pJu4014	228A cosmid derivative where *gcnG1* was disrupted by *aac(3)IV* + *oriT* using primers G1delF and G1delR	This study
pJu4015	228A cosmid derivative where *gcnG2* was disrupted by aac(3)IV + oriT using primers G2delF and G2delR	This study
pJu4016	228A cosmid derivative where *gcnG1G2* was disrupted by aac(3)IV + oriT using primers G1delF and G2delR	This study
pJu4017	228A cosmid derivative where *gcnG3* was disrupted by aac(3)IV + oriT using primers G3delF and G3delR	This study

Table S3. The primer pairs used for mutant construction.

Gene target	Primer code	Primer pairs used for inactivation (5′-3′)
gcnG1	G1delF	GTCCTGTTCACCATCTTTCCGGCGACGGCGCACCTGTATattccggggatccgtcgacc
	G1delR	GACCATCTCGTCCCACATGACCAGCGGCATCACCAGCTGtgtaggctggagctgcttc
gcnG2	G2delF	TTCGAGGTGAACGGGATGCTGCTGGAGGGGCTGGAGCCGattccggggatccgtcgacc
	G2delR	CAGCGGGAAGTGGTCCGCCAGCTGCGGAAGCACCAACTGtgtaggctggagctgcttc
gcnG1 G2	G1delF	GTCCTGTTCACCATCTTTCCGGCGACGGCGCACCTGTATattccggggatccgtcgacc
	G2delR	CAGCGGGAAGTGGTCCGCCAGCTGCGGAAGCACCAACTGtgtaggctggagctgcttc
gcnG3	G3delF	ACCACCGATCTGCCGATCCGGCACTTCATCACCACCGACattccggggatccgtcgacc
	G3delR	CTTGGGAATGAGGAGTTGCGGTACACCGGCGTTCAGACCtgtaggctggagctgcttc

Table S4. The primers used for PCR confirmation of the double-crossover mutants.

Gene	Primer code	Primer pairs designed to verify the mutant strains (5′-3′)	Fragment replaced	Length of desired PCR fragments	
				Wild strain	Mutant strain
gcnG1	G1tF	CTTCGCAGCCCTCAATCTCCA	999	1449	1819
	G1tR	GTACGAGCTGCTTGTGCATCT			
gcnG2	G2tF	ACCCATTTCCTTCCGTTGGTG	717	1035	1687
	G2tR	GTCGGACAGCAGCTCGTCCAG			
gcnG1G2	G1tF	CTTCGCAGCCCTCAATCTCCA	2201	2668	1836
	G2tR	GTCGGACAGCAGCTCGTCCAG			
gcnG3	G3tF	ATGGTCCCGTACATCACGTCG	687	1044	1726
	G3tR	TCCTCTCGGTTCGTGTCGTCT			

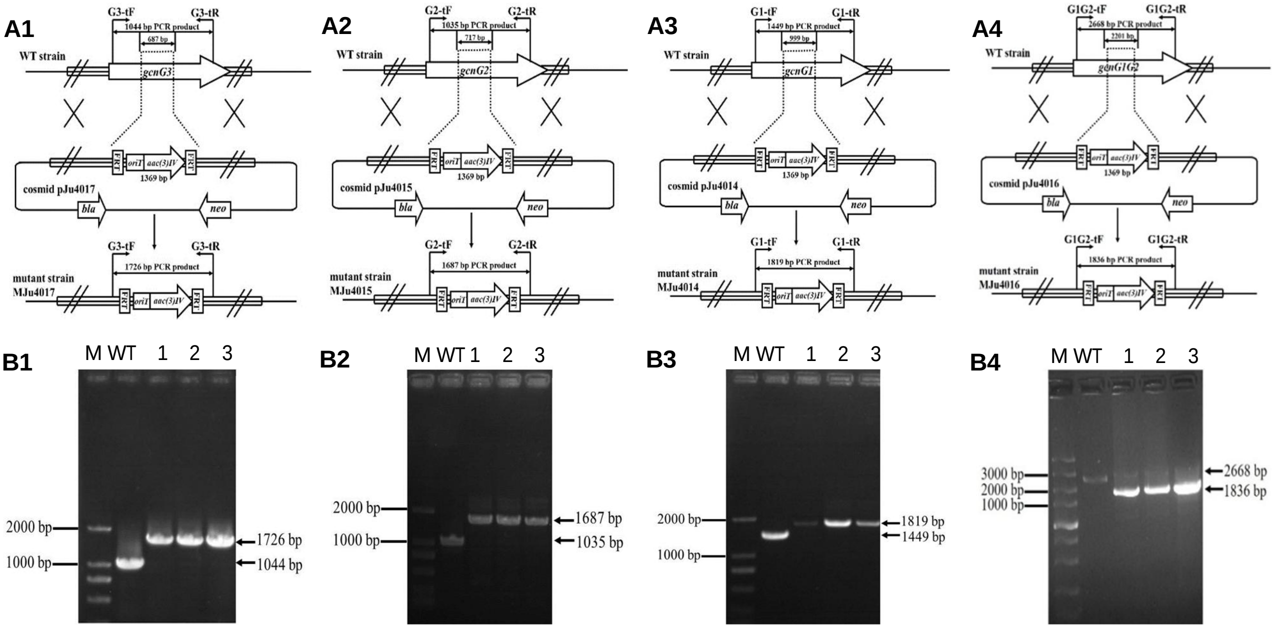

Figure S1. Gene inactivation of *gcnG3~gcnG1*, and *gcnG1G2*. A1~A4: construction of Δ*gcnG3*, Δ*gcnG2*, Δ*gcnG1*, and Δ*gcnG1G2* mutants; B1~B4: gel electrophoresis of PCR products, DNA templates were from DNA marker DL 2000 (Takara, lane M), *Streptomyces lusitanus* SCSIO LR32 (lane WT), and Δ*gcnG3*, Δ*gcnG2*, Δ*gcnG1*, and Δ*gcnG1G2* mutant strains (lanes 1, 2, 3), respectively.

Figure S2. Procedure for isolation of compound from the mutant Δ*gcnG3*.

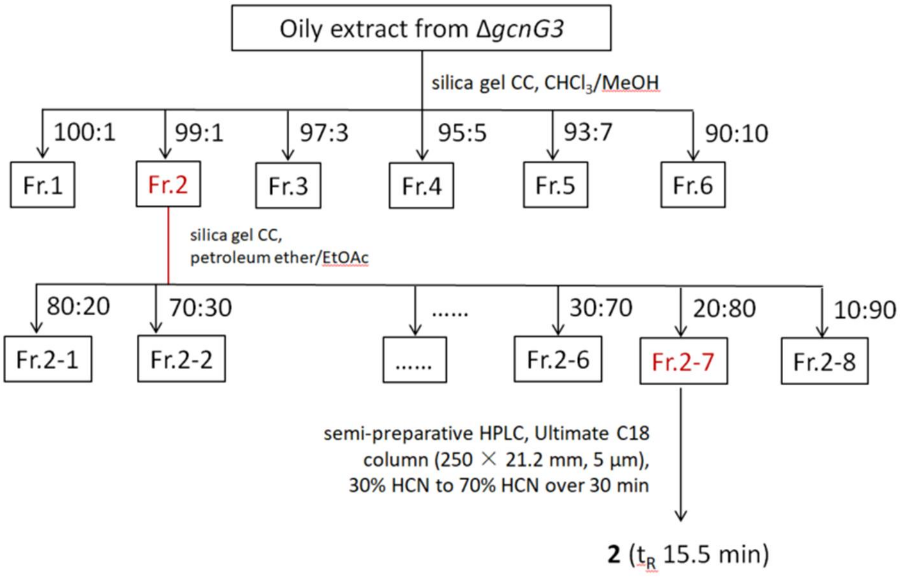

Figure S3. Procedure for isolation of compounds from the mutant Δ*gcnG2*.

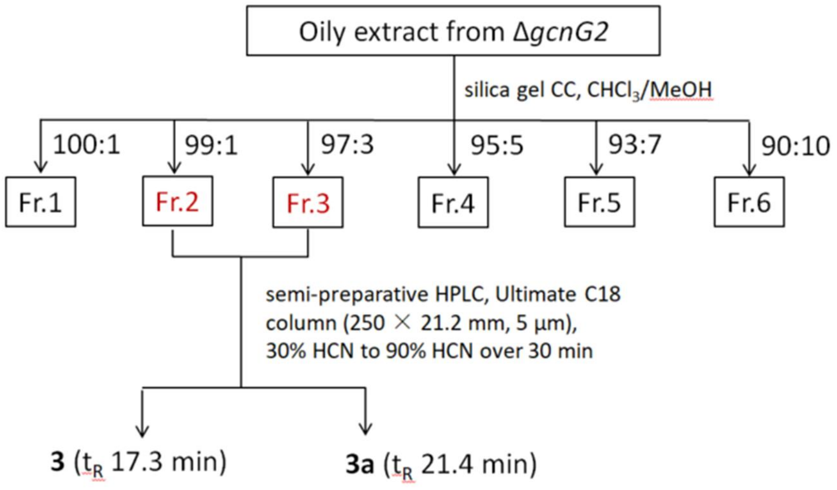

Figure S4. Procedure for isolation of compound from the mutant Δ*gcnG1*.

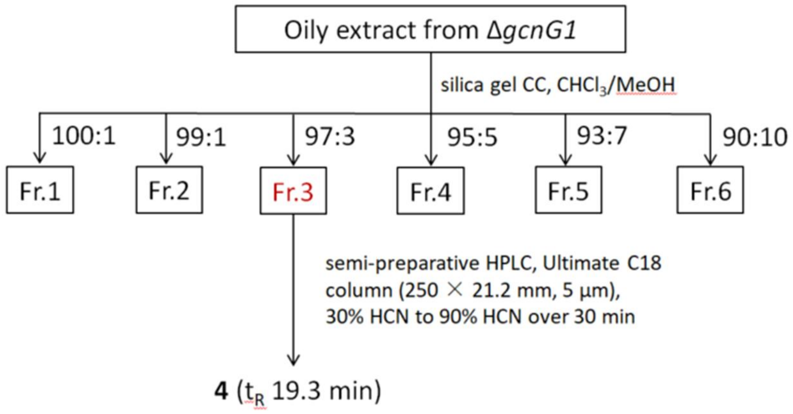

Figure S5. Procedure for isolation of compound from the mutant Δ*gcnG1G2*.

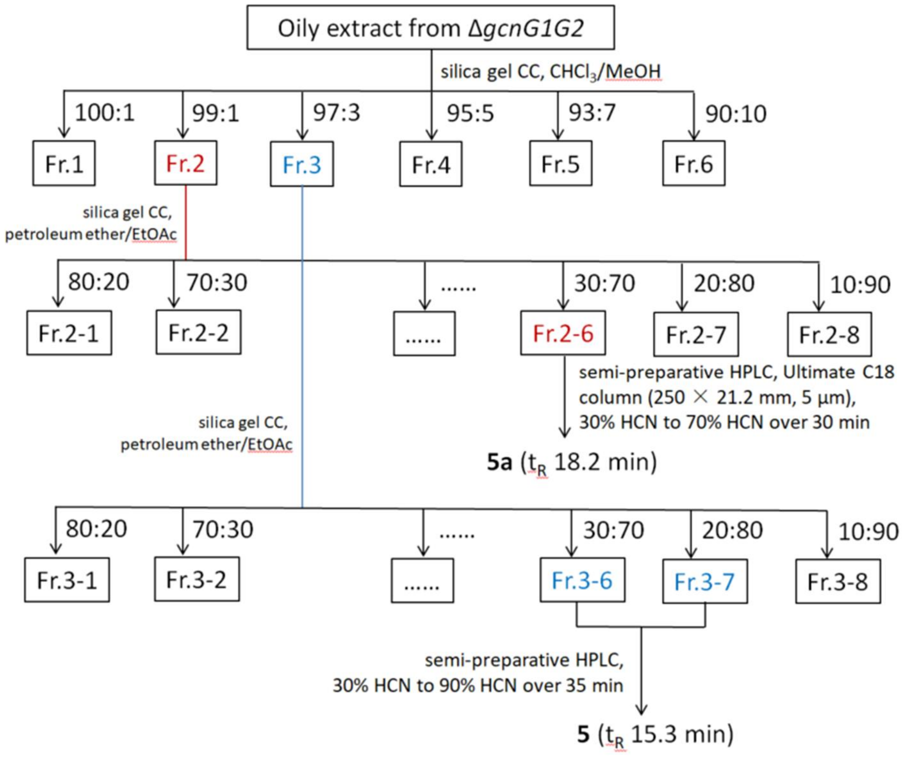

Figure S6. The UV spectrum of grincamycin U (**3a**).

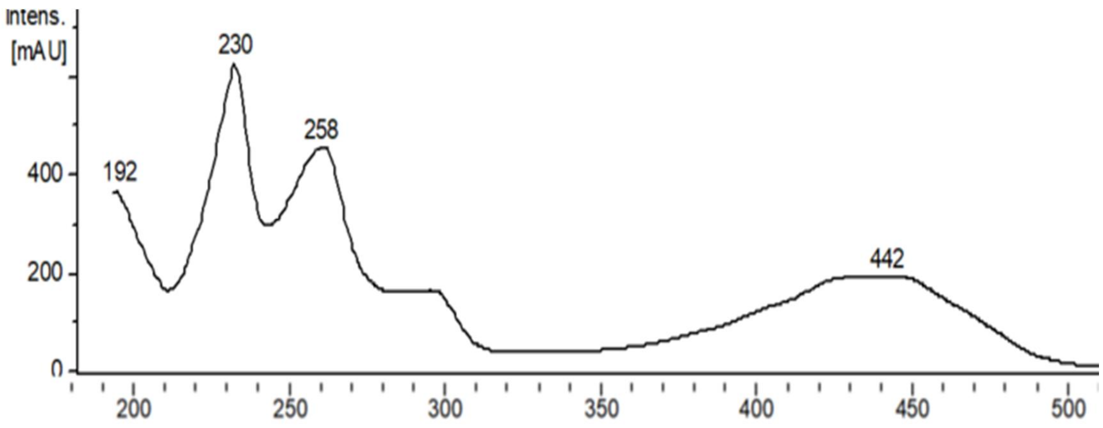

Figure S7. The (-)HR-ESI-MS spectrum of grincamycin U (**3a**).

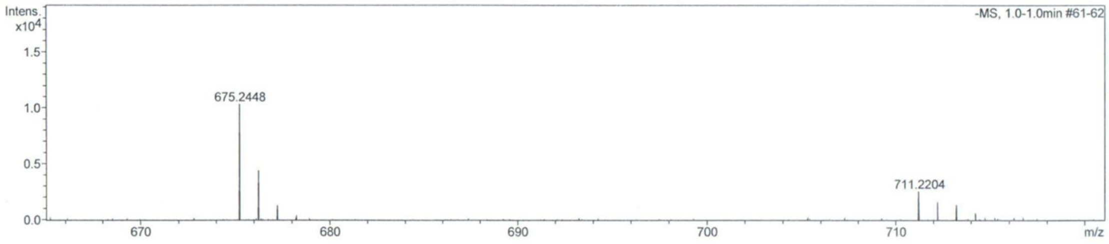

Meas. m/z	#	Ion Formula	Score	m/z	err [ppm]	err [mDa]	mSigma	rdb	e⁻ Conf	N-Rule
675.2448	1	C37H39O12	100.00	675.2447	0.1	0.1	15.8	18.5	even	ok
711.2204	1	C37H40ClO12	100.00	711.2214	1.4	1.0	111.9	17.5	even	ok
1351.4981	1	C74H79O24	100.00	1351.4967	-1.1	-1.5	71.5	35.5	even	ok

Figure S8. The ^{1}H NMR (700 MHz) spectrum of grincamycin U (**3a**) in CD_3OD.

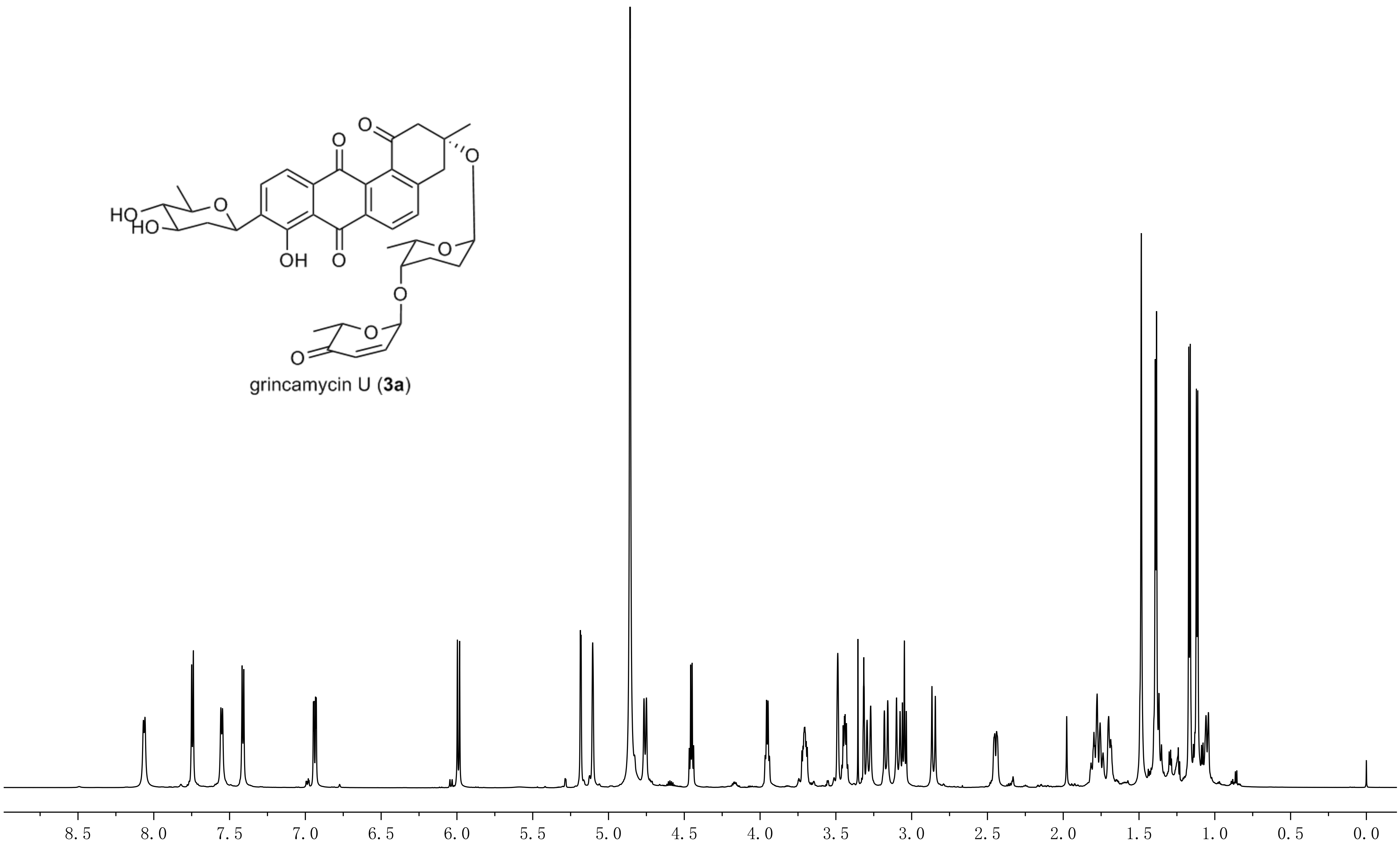

Figure S9. The ^{13}C NMR (175 MHz) spectrum of grincamycin U (**3a**) in CD_3OD.

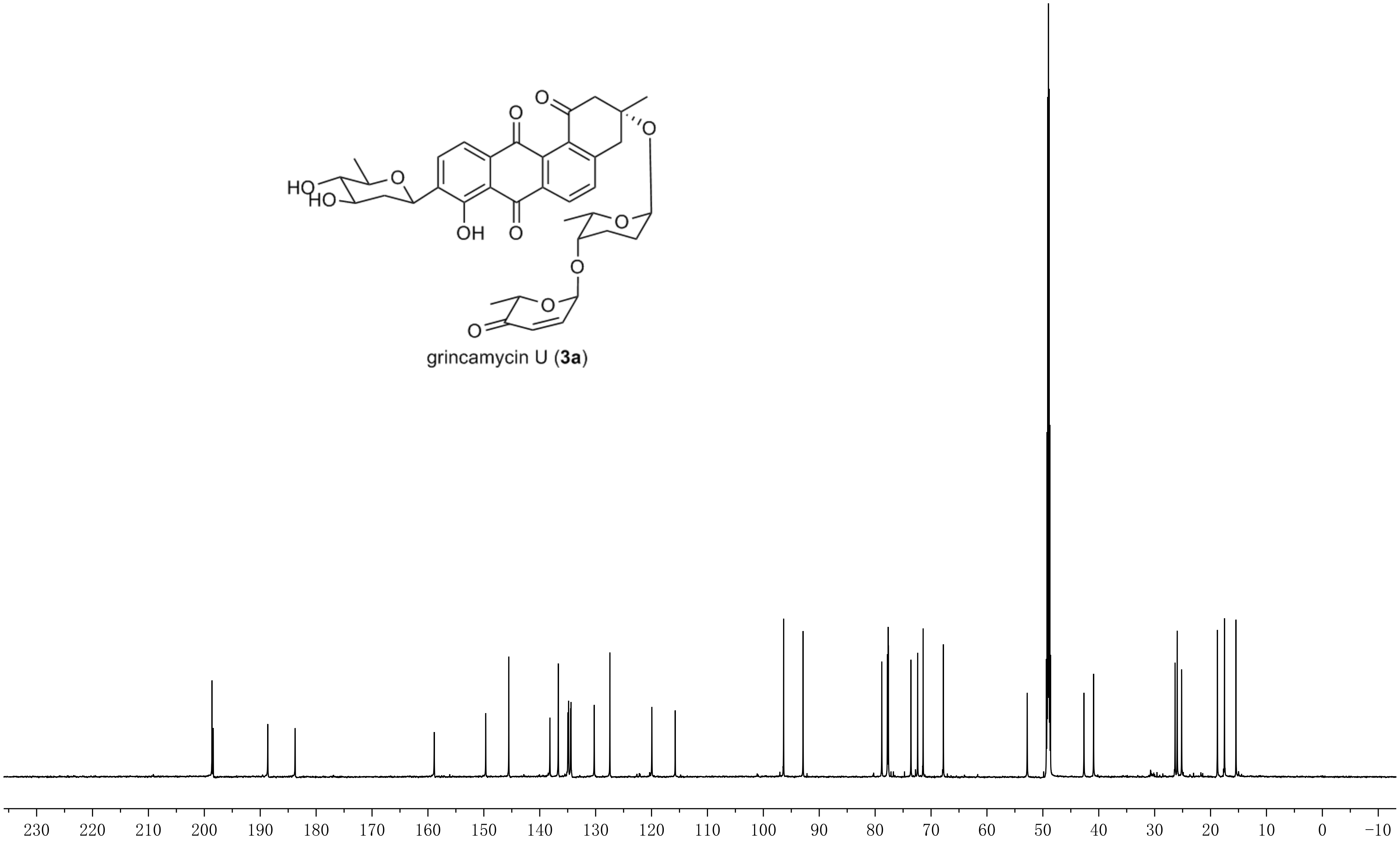

Figure S10. The DEPT-135 NMR (175 MHz) spectrum of grincamycin U (**3a**) in CD_3OD.

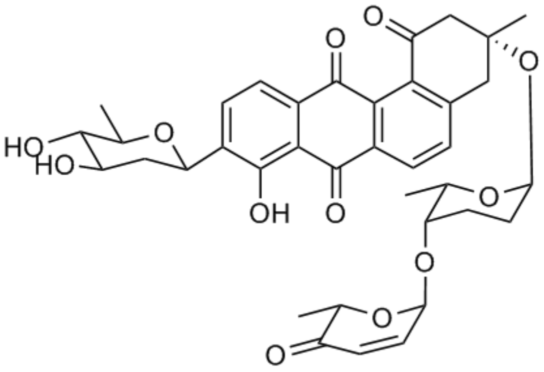

grincamycin U (**3a**)

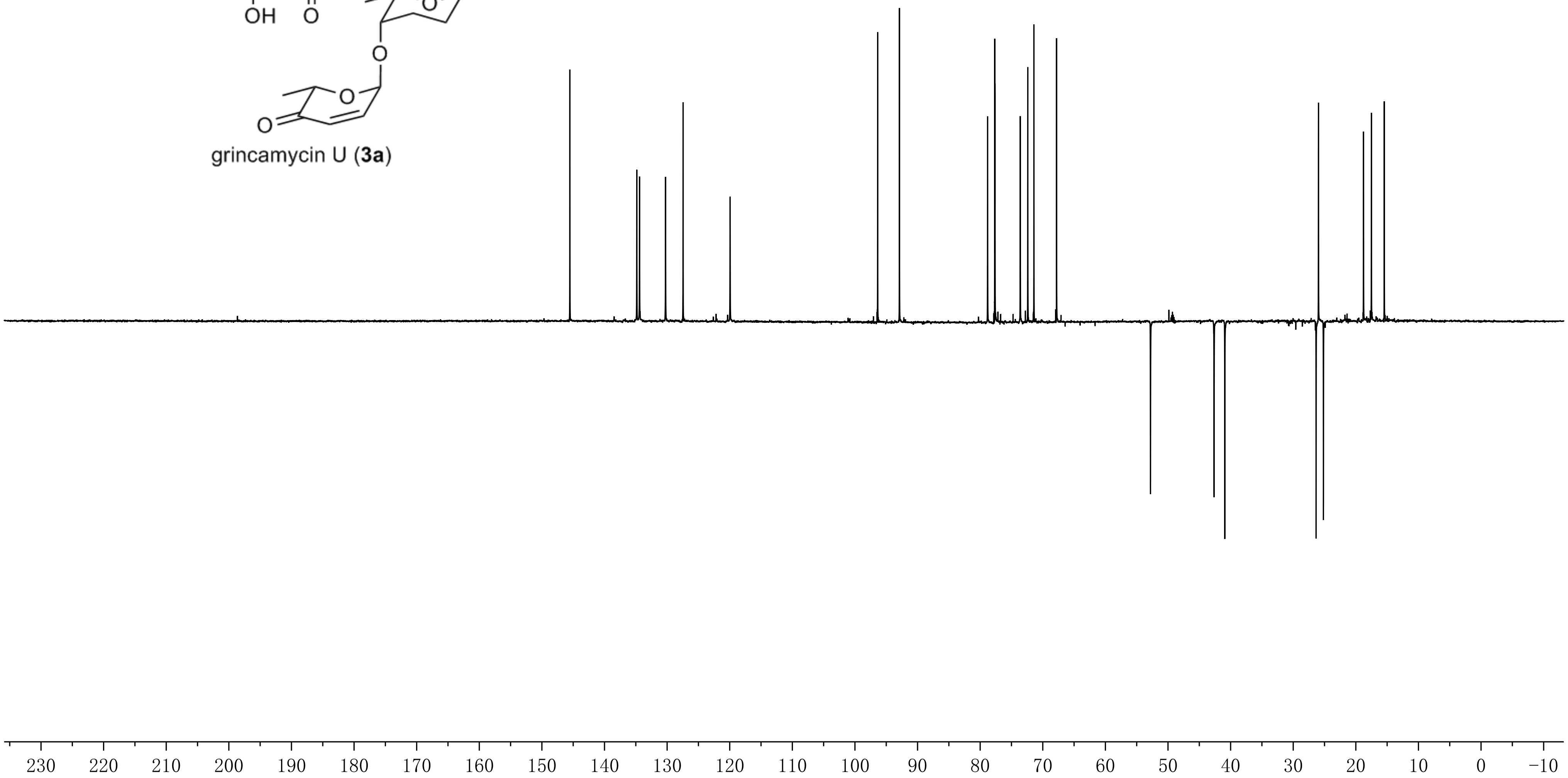

Figure S11. The COSY NMR spectrum of grincamycin U (**3a**) in CD_3OD.

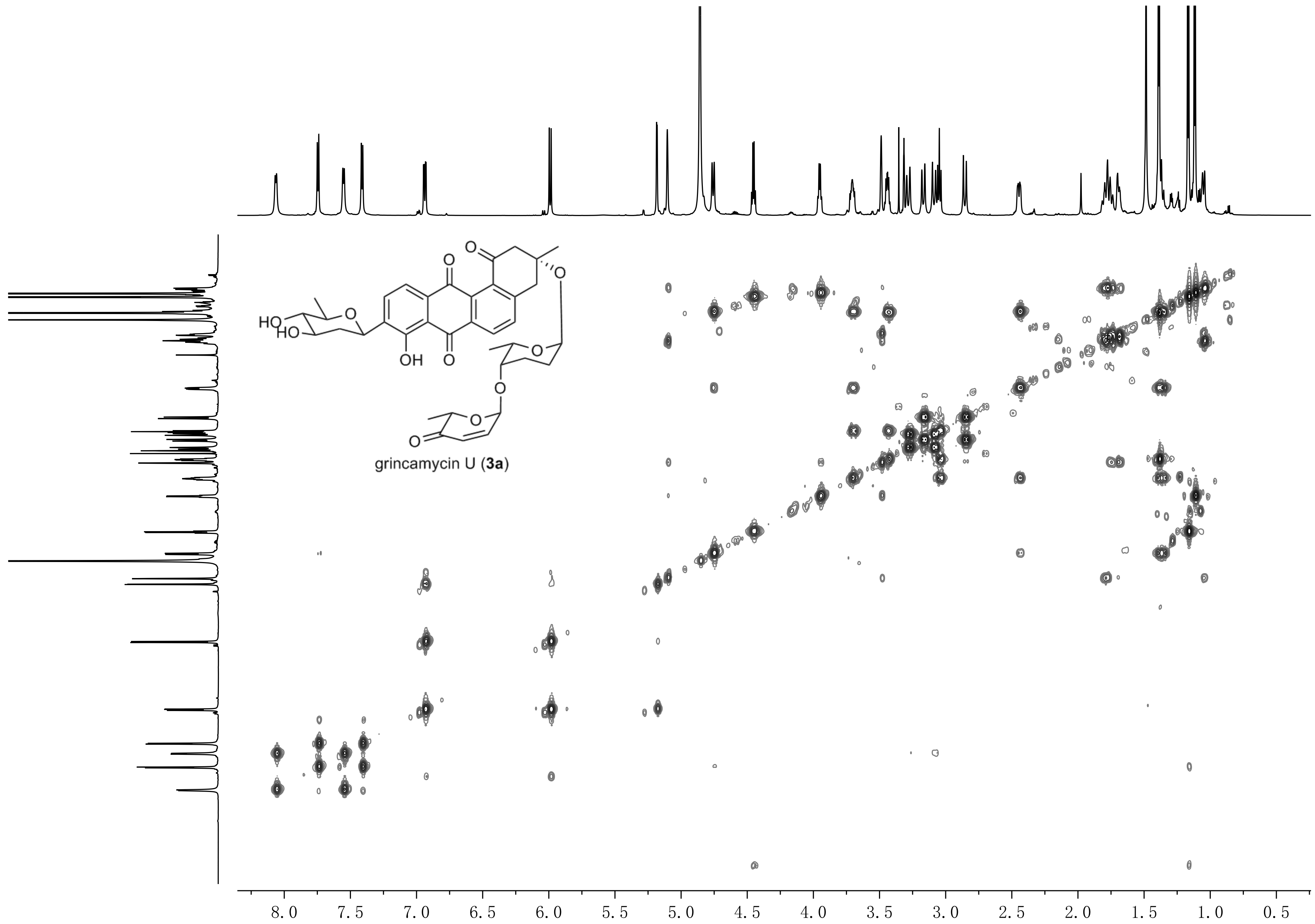

Figure S12. The HMQC NMR spectrum of grincamycin U (**3a**) in CD_3OD.

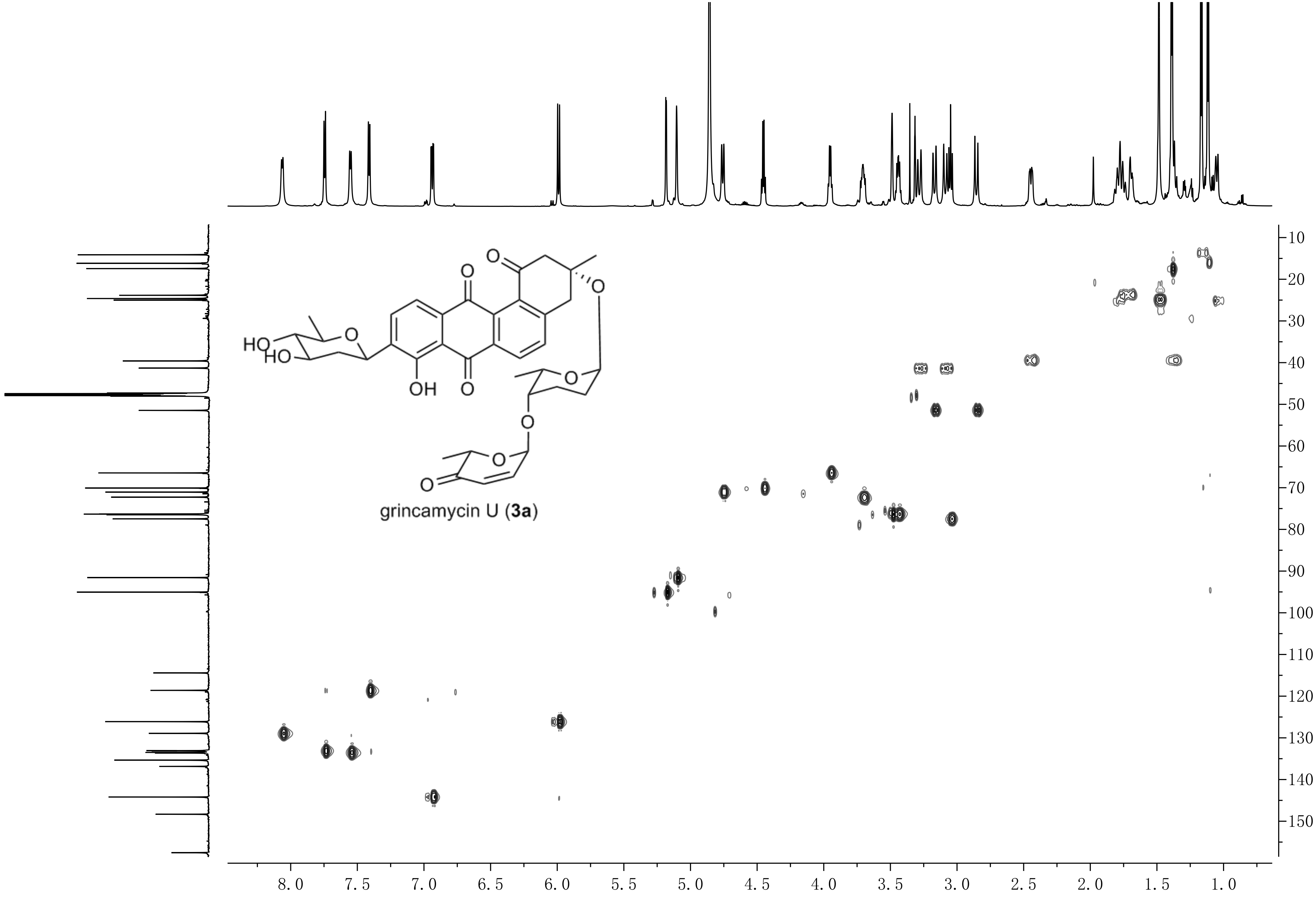

Figure S13. The HMBC NMR spectrum of grincamycin U (**3a**) in CD_3OD.

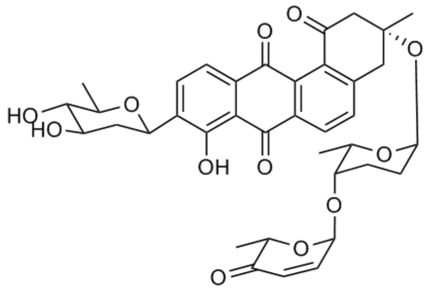

grincamycin U (**3a**)

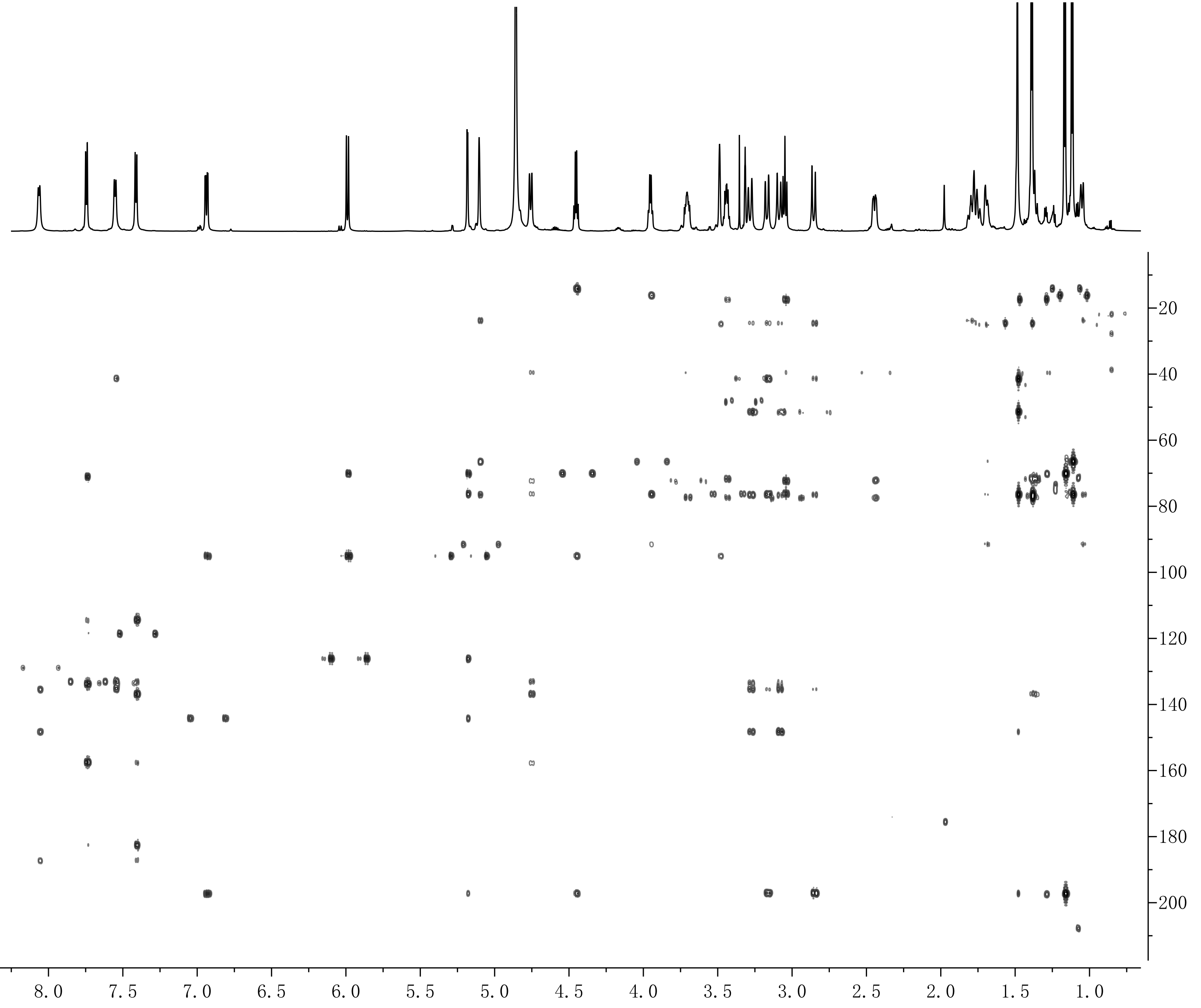

Figure S14. The NOESY NMR spectrum of grincamycin U (**3a**) in CD_3OD.

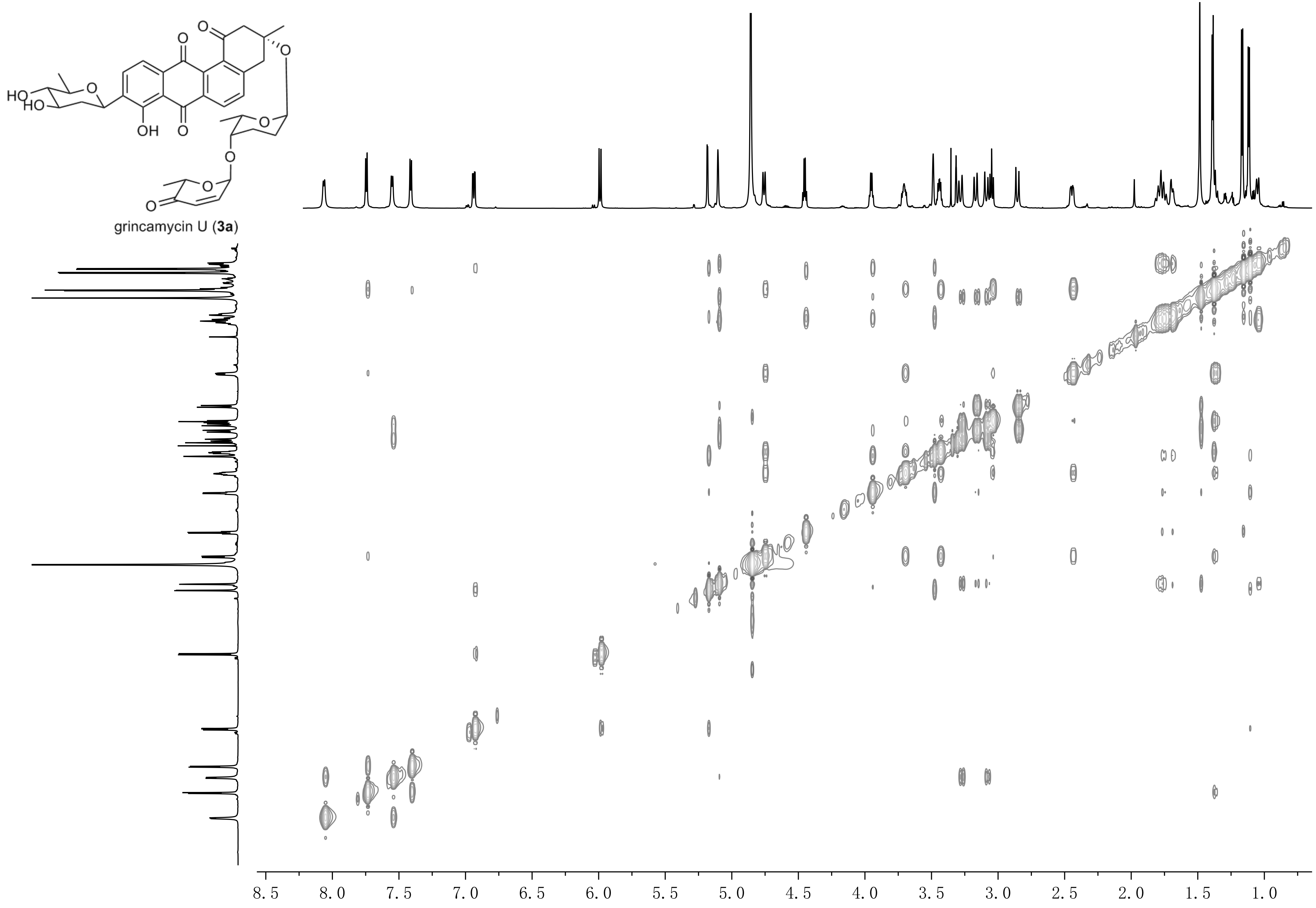

Figure S15. The UV spectrum of grincamycin V (**3'**).

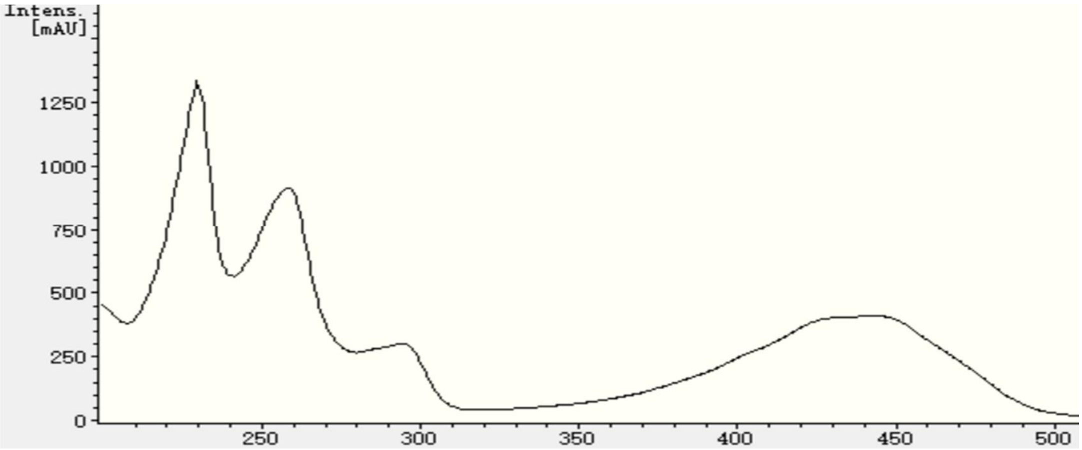

Figure S16. The (-)HR-ESI-MS spectrum of grincamycin V (**3'**).

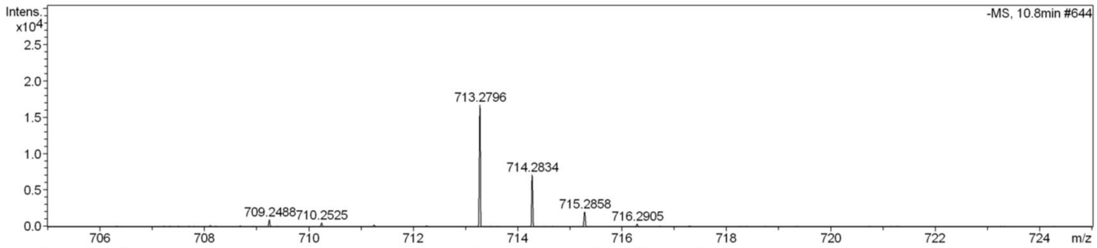

Meas. m/z	#	Ion Formula	Score	m/z	err [ppm]	err [mDa]	mSigma	rdb	e⁻ Conf	N-Rule
713.2796	1	C37H45O14	100.00	713.2815	-2.6	-1.9	9.9	15.5	even	ok

Figure S17. The ^{1}H NMR (700 MHZ) spectrum of grincamycin V (**3'**) in CD_3OD.

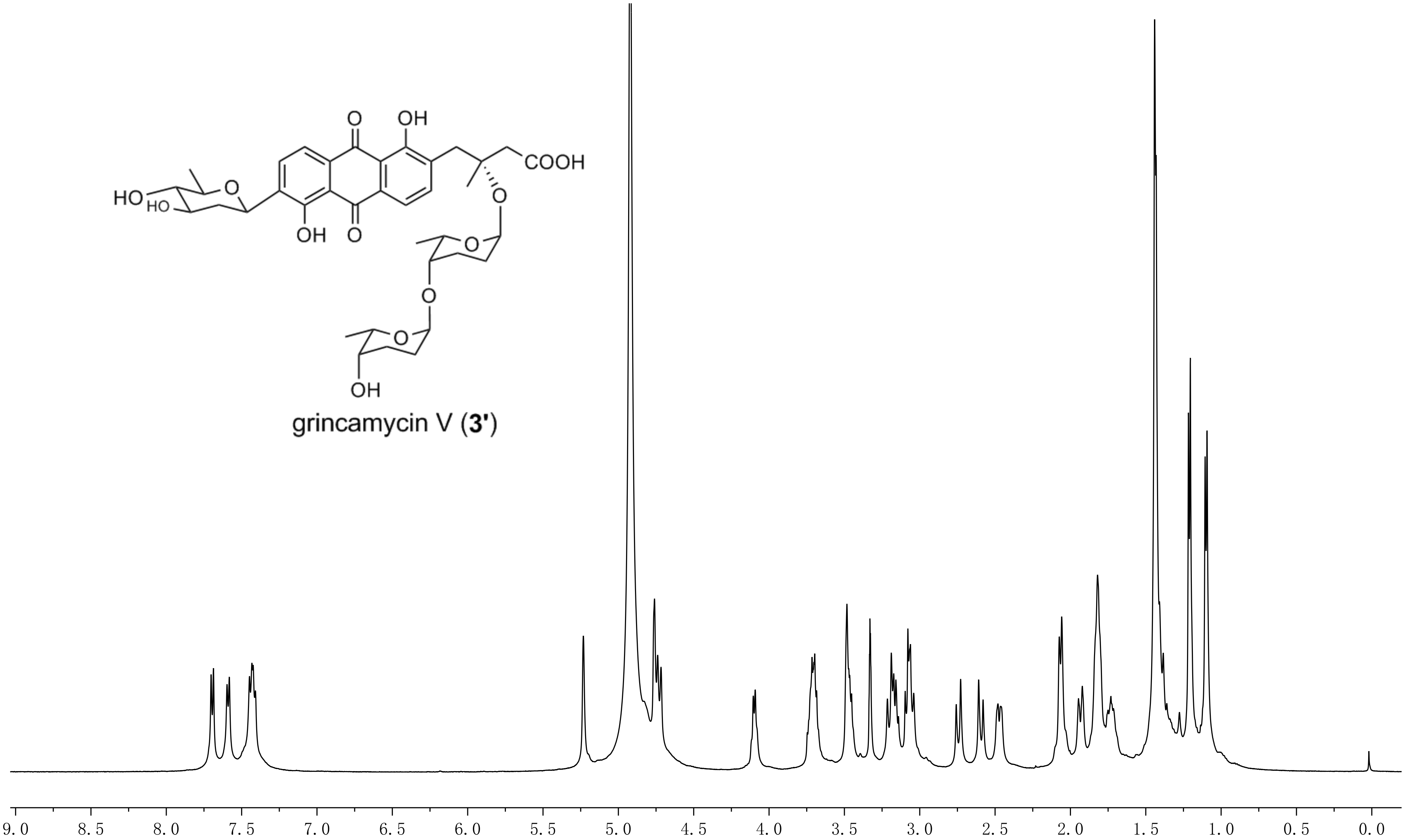

Figure S18. The ^{13}C NMR (175 MHz) spectrum of grincamycin V (**3'**) in CD_3OD.

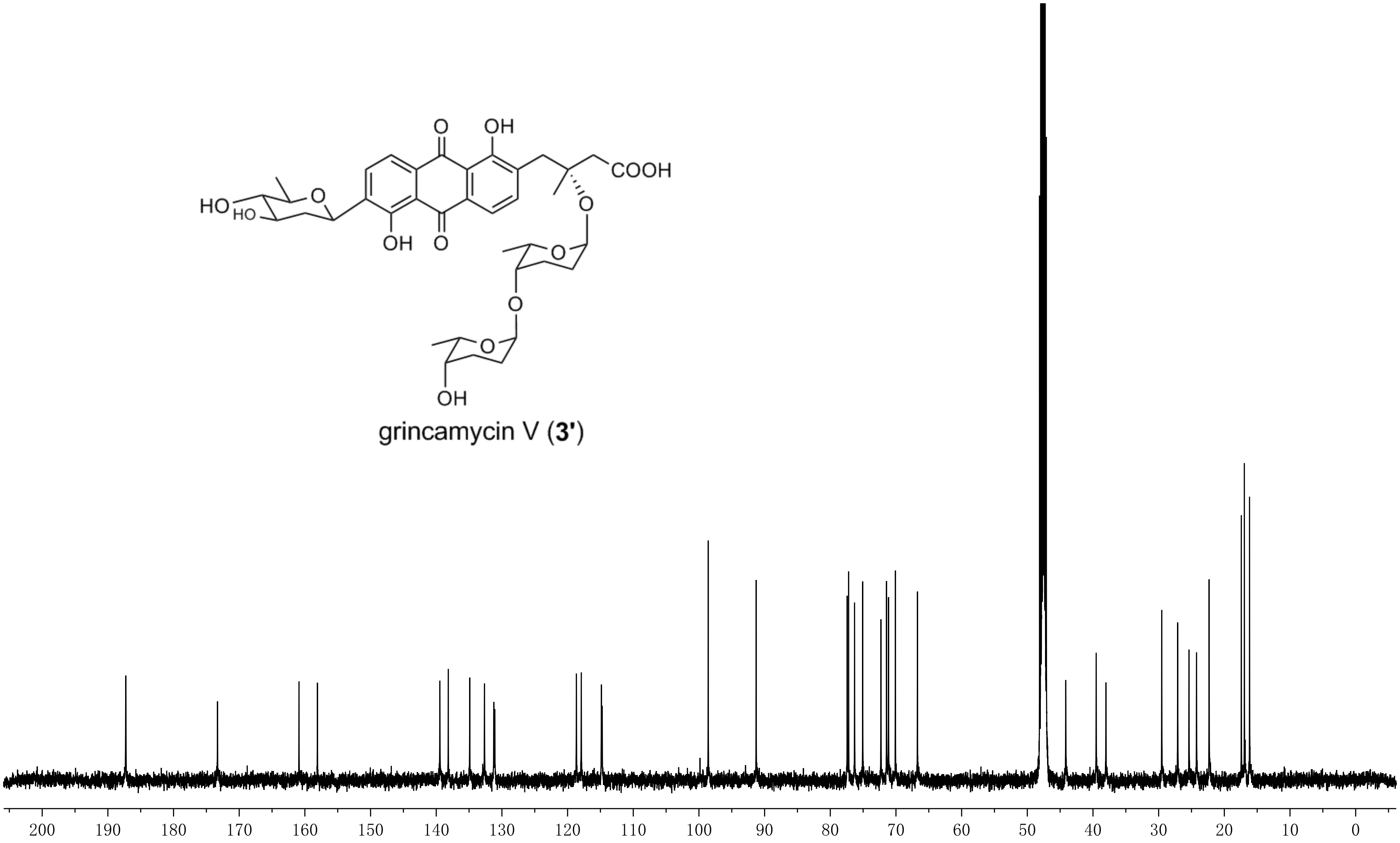

Figure S19. The DEPT-135 NMR spectrum of grincamycin V (**3'**) in CD_3OD.

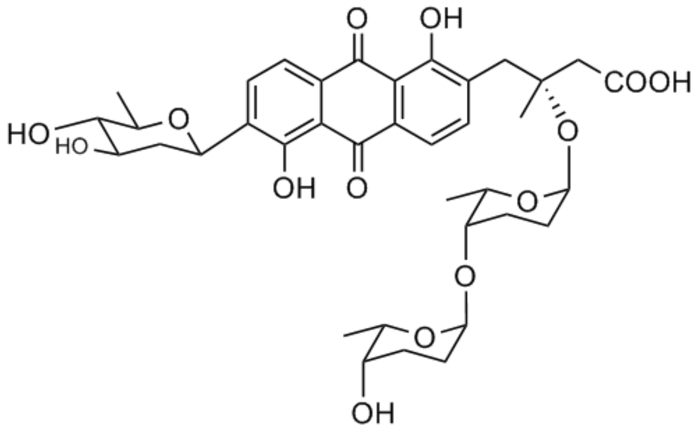

grincamycin V (**3'**)

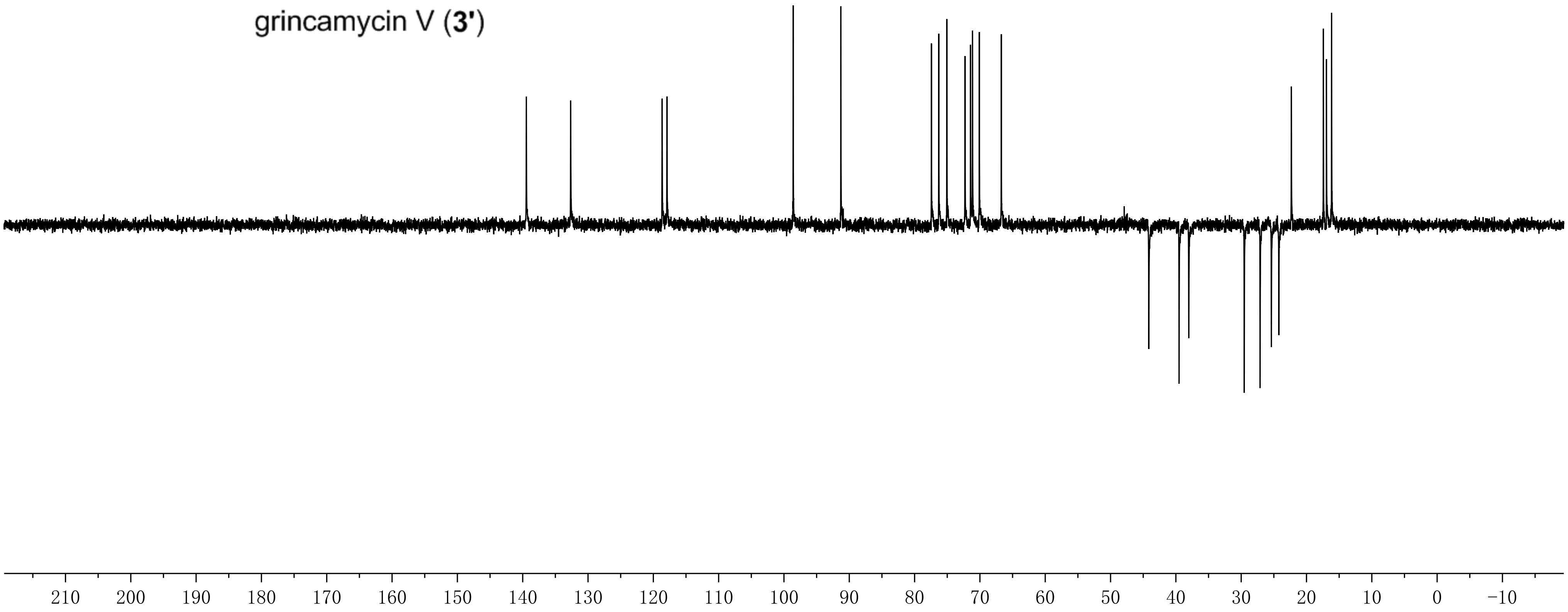

Figure S20. The NOESY NMR spectrum of grincamycin V (**3'**) in CD_3OD.

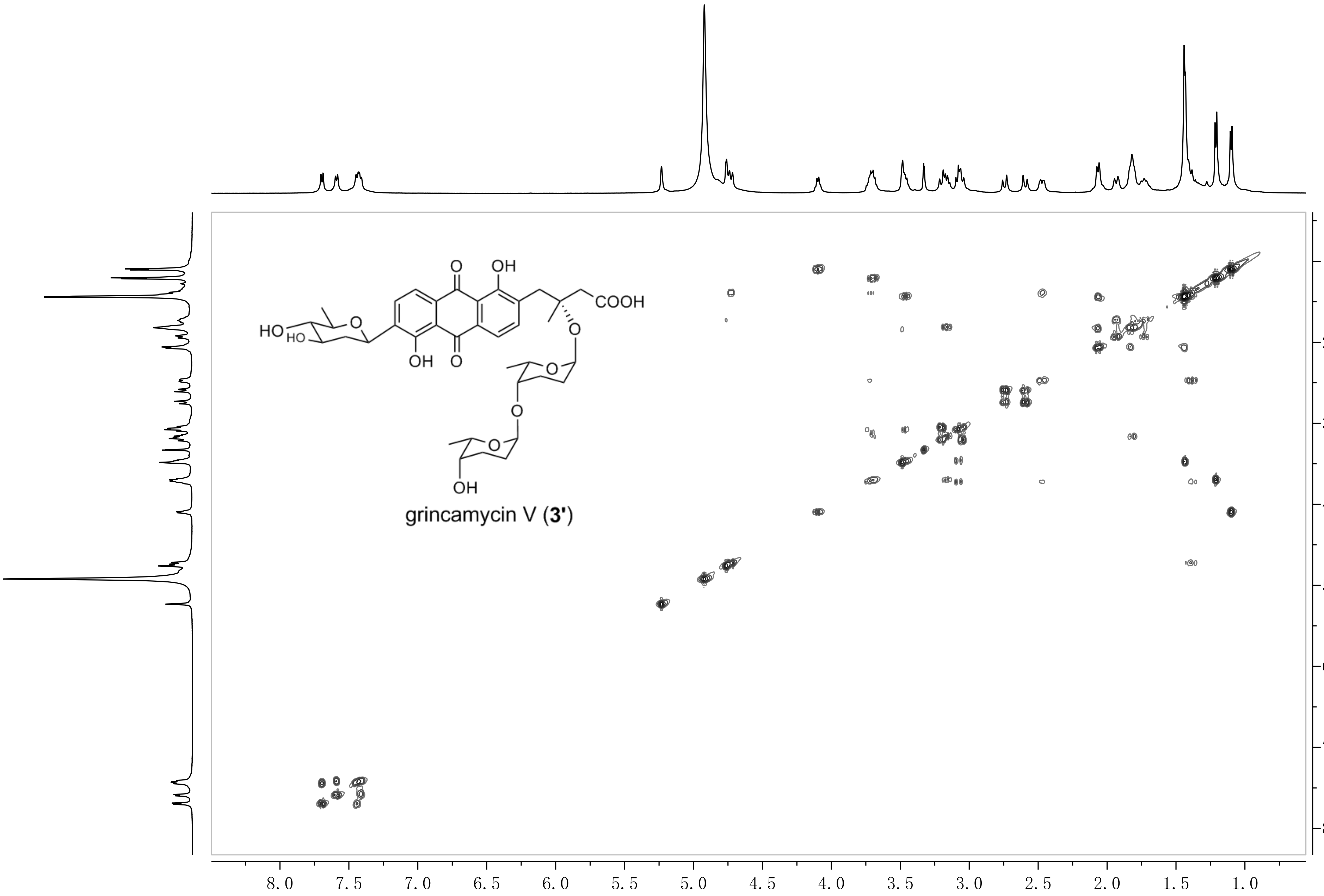

Figure S21. The HMQC NMR spectrum of grincamycin V (**3'**) in CD_3OD.

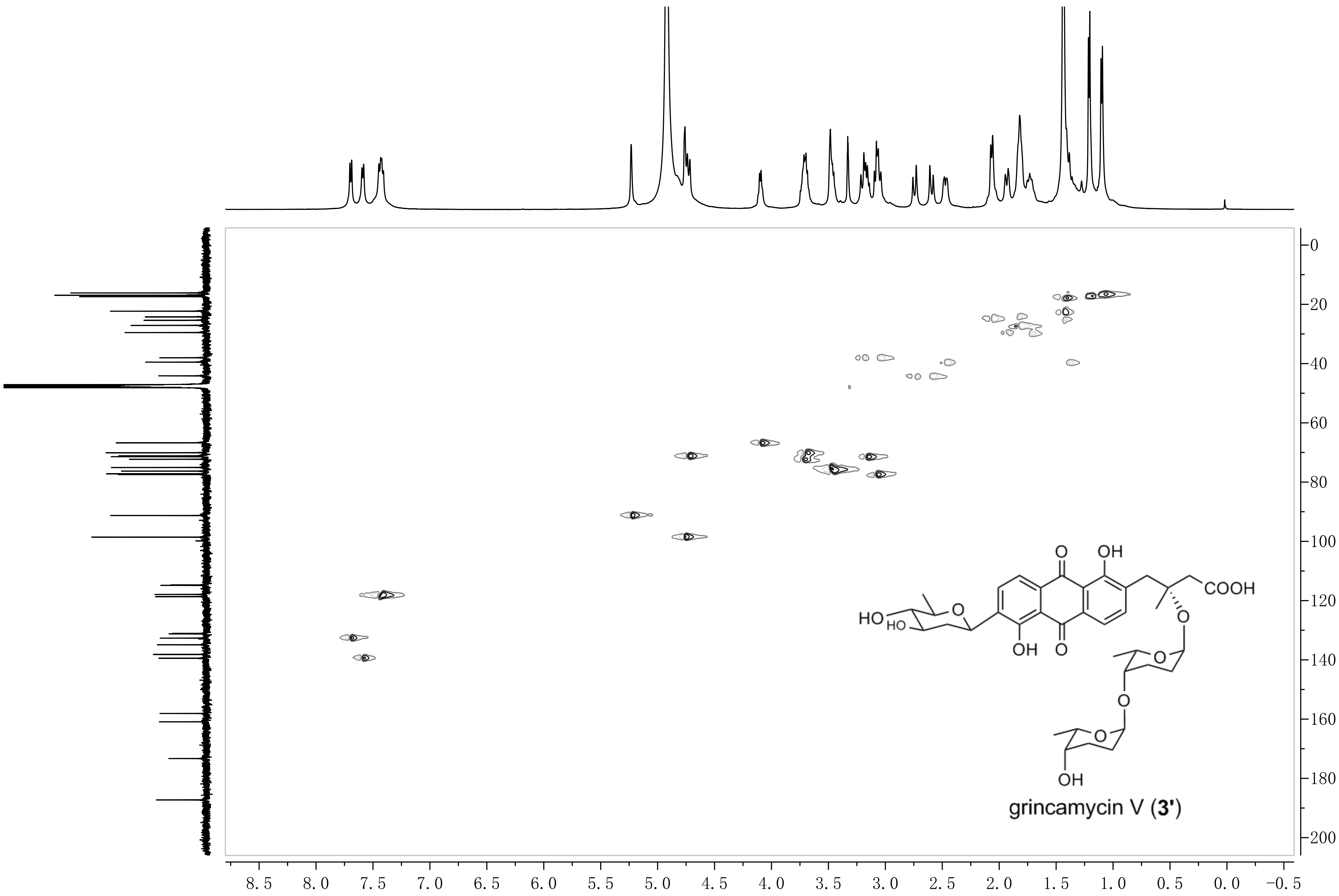

Figure S22. The HMBC NMR spectrum of grincamycin V (**3'**) in CD_3OD.

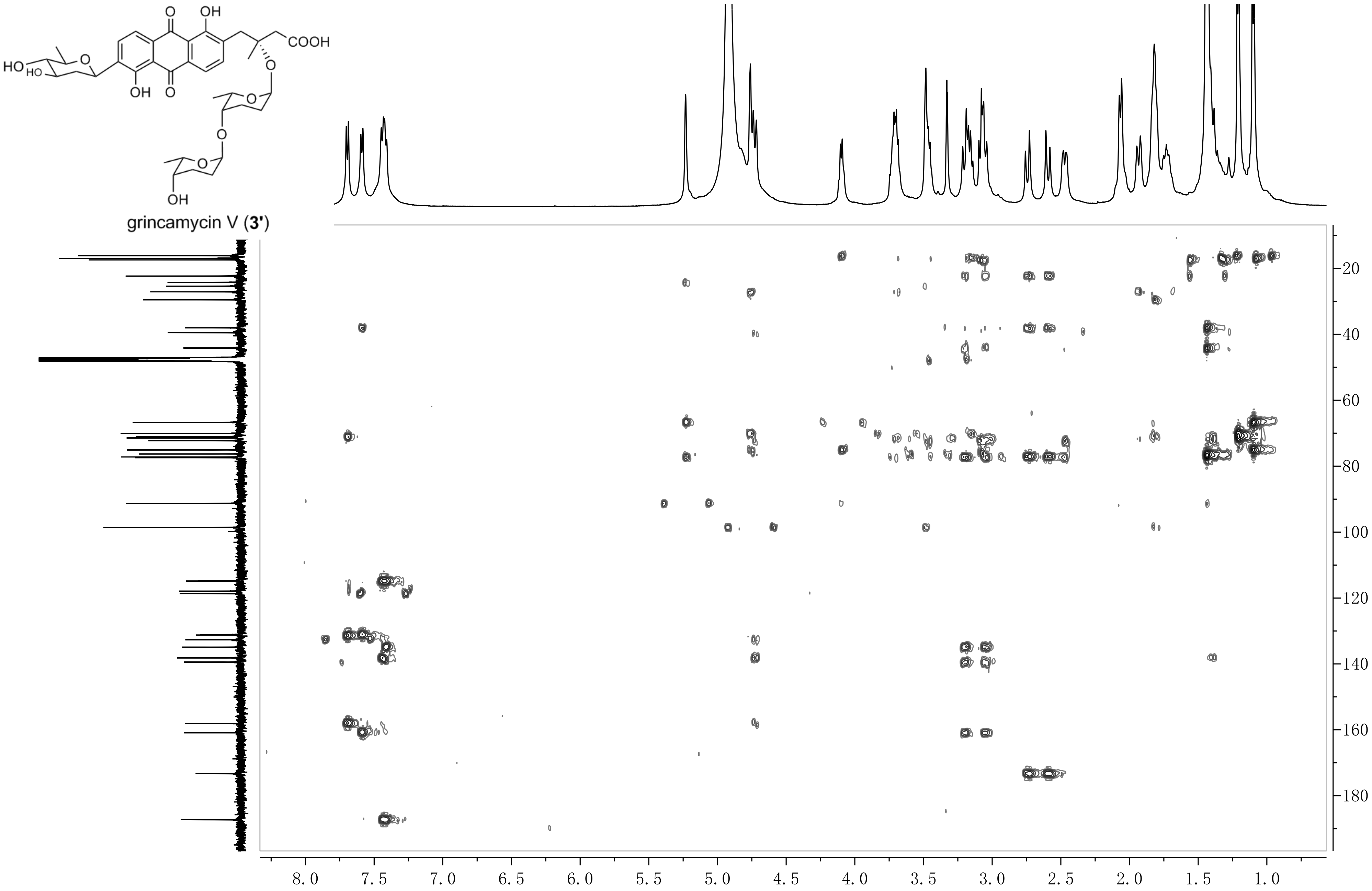

Figure S23. The NOESY NMR spectrum of grincamycin V (**3'**) in CD_3OD.

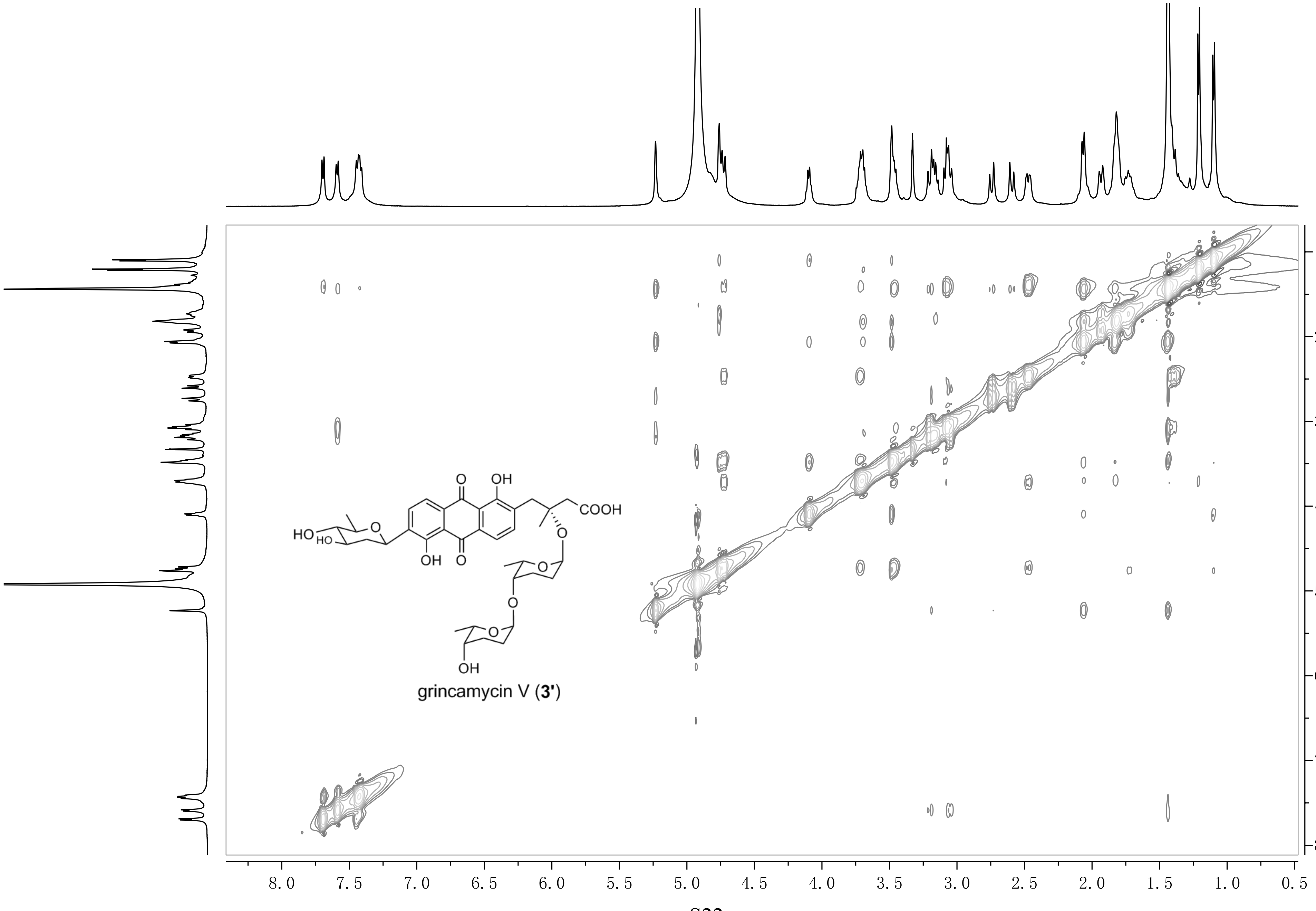

Supplement: Supplementary file 1 [file marinedrugs-22-00032-s001.zip › marinedrugs-2794782-supplementary.pdf]
